# Exceptionally High Perfluorooctanoic Acid Uptake in Water by a Zirconium-Based Metal–Organic Framework through Synergistic Chemical and Physical Adsorption

**DOI:** 10.1021/jacs.3c14487

**Published:** 2024-03-26

**Authors:** Rong-Ran Liang, Shunqi Xu, Zongsu Han, Yihao Yang, Kun-Yu Wang, Zhehao Huang, Joshua Rushlow, Peiyu Cai, Paolo Samorì, Hong-Cai Zhou

**Affiliations:** † Department of Chemistry, Texas A&M University, College Station, Texas 77843, United States; ‡ Université de Strasbourg, CNRS, ISIS, 8 alleé Gaspard Monge, 67000 Strasbourg, France; § Department of Materials and Environmental Chemistry, Stockholm University, SE-106 91 Stockholm, Sweden

## Abstract

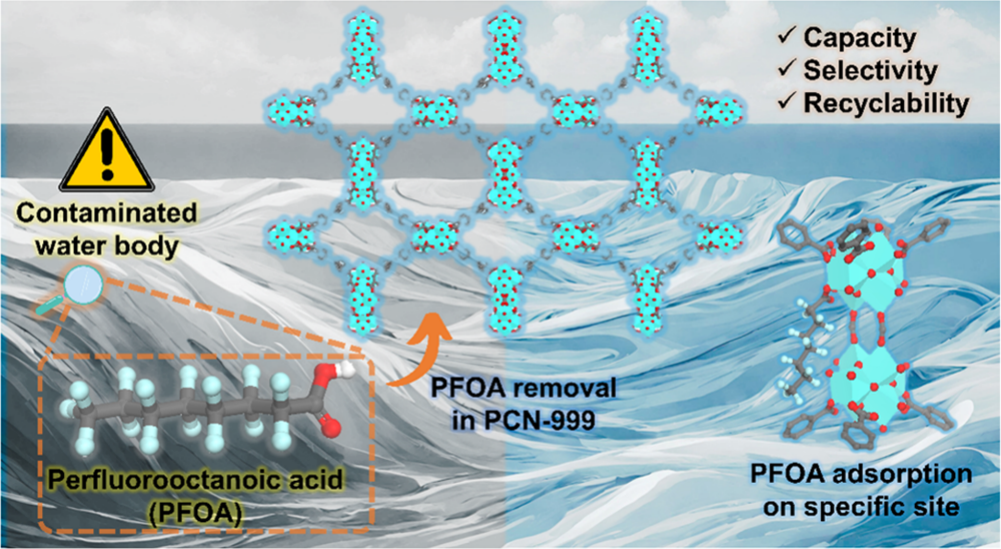

Perfluorooctanoic acid (PFOA) is an environmental contaminant ubiquitous in water resources, which as a xenobiotic and carcinogenic agent, severely endangers human health. The development of techniques for its efficient removal is therefore highly sought after. Herein, we demonstrate an unprecedented zirconium-based MOF (PCN-999) possessing Zr_6_ and biformate-bridged (Zr_6_)_2_ clusters simultaneously, which exhibits an exceptional PFOA uptake of 1089 mg/g (2.63 mmol/g), representing a ca. 50% increase over the previous record for MOFs. Single-crystal X-ray diffraction studies and computational analysis revealed that the (Zr_6_)_2_ clusters offer additional open coordination sites for hosting PFOA. The coordinated PFOAs further enhance the interaction between coordinated and free PFOAs for physical adsorption, boosting the adsorption capacity to an unparalleled high standard. Our findings represent a major step forward in the fundamental understanding of the MOF-based PFOA removal mechanism, paving the way toward the rational design of next-generation adsorbents for per- and polyfluoroalkyl substance (PFAS) removal.

## Introduction

Per- and polyfluoroalkyl substances (PFASs) are an emerging class of highly recalcitrant pollutants characterized by hydrophobic fluorinated carbon chains and hydrophilic terminal functional groups. The energy of carbon–fluorine (C–F) bonds, amounting to 488 kJ/mol, is the highest among covalent linkages in organic chemistry, resulting in the PFASs’ exceptional thermal and chemical stability for diverse applications ranging from aqueous film-forming foams and waterproof fabrics to electrical wires coverings.^1,2^ However, the strong C–F bonds also make PFASs “forever chemicals”, resulting in their bioaccumulation in both the environment and the human body,^3,4^ ultimately significantly increasing the risk of liver cancer and immune response suppression. Among all PFAS compounds, perfluorooctanoic acid (PFOA) displays high water solubility (9.5 g/L), and it has been classified as “possibly carcinogenic to humans” by the International Agency for Research on Cancer (IARC) due to the strong correlation between PFOA exposure and testicular and kidney cancer.^5^

So far, extensive efforts have been devoted to PFAS removal from contaminated water using different strategies including adsorption,^3,6−8^ photocatalysis,^9^ electrochemical oxidation,^10,11^ and biological remediation.^12,13^ Among these methodologies, adsorption stands out, owing to its simple operation, efficiency, and cost-effectiveness. Currently, activated carbon and ion-exchange resin are the state-of-the-art adsorbents for PFAS removal.^3^ Nevertheless, they suffer from limited efficiency and poor regenerability. Hence, the development of novel and long-lasting adsorbents combining high selectivity and a high capacity to sequester PFOA is urgently needed.

Metal–organic frameworks (MOFs) are a class of porous crystalline materials^14,15^ consisting of organic ligands and metal-containing secondary building units (SBUs), which are assembled through coordination bonds.^16−22^ Properties of MOFs can be programmed via ad hoc chemical synthesis yielding high surface area, extensive porosity, tunable structures, and diverse functionalities, which make them ideal candidates for eliminating pollutants from contaminated water with high adsorption selectivity/capacity and rapid kinetics. Compared to other porous materials such as amorphous porous organic polymers^23,24^ and covalent organic frameworks,^6,25^ MOFs are known for their exceptional porosities and high crystallinity, which render them ideal adsorbents for PFAS adsorption. Although several well-known MOFs, such as MIL-101, ZIF-8, UiO-66, and NU-1000, have been reported for PFAS removal,^26−28^ the achievement of both high adsorption capacity and unambiguous elucidation of the adsorption mechanism remains a significant challenge. In this context, the development of robust MOFs with a suitable pore environment and PFOA-binding site (*e.g*. open coordination site) is highly desired. In addition, subsequent to the PFOA-metal coordination, the chemically modified internal surface of the pores may act as a seed for the clustering of further PFAS contaminants via physical adsorption, collectively boosting the overall uptake of binary and multicomponent PFAS contaminants.

In this work, we present the synthesis of a novel zirconium-based MOF (PCN-999) bearing two types of metal-containing SBUs, namely, Zr_6_ and biformate-bridged (Zr_6_)_2_ clusters, constructed through linker desymmetrization.^29−31^ (Zr_6_)_2_ SBU comprises two Zr_6_ clusters bridged by two formates, which exclusively connect to the short arms of the desymmetrized ligand. To the best of our knowledge, for the first time, such an eight-connected (Zr_6_)_2_ SBU has been incorporated into a MOF skeleton, thereby generating additional open coordination sites to host PFOA. Moreover, PCN-999 displays a hierarchically porous architecture, which consists of one-dimensional (1D) hexagonal mesoporous channels (∼22 Å) and 1D rhombic microporous channels (∼12 Å) along the *a* axis. Notably, the synergistic effect of the open coordination sites and the hierarchically porous architecture as well as the remarkable hydrolytic, thermal, and chemical stabilities^32^ creates an unprecedented PFOA adsorption capacity (1089 mg/g, 2.63 mmol/g) in water. This remarkable adsorption capacity is accompanied by rapid adsorption kinetics, long cycle life, and high selectivity. Detailed structural analyses, including single-crystal X-ray diffraction (SCXRD), Fourier-transform infrared (FTIR) spectroscopy, porosity tests, and computations revealed the coordination of the carboxylate group of PFOA exclusively with the (Zr_6_)_2_ SBU and the ’zigzag’ alignment of the coordinated PFOA within the mesoporous channels of PCN-999. Moreover, the coordinated PFOA acts as a seed for the clustering process, which occurs through the physical adsorption of free PFOA onto previously coordinated ones. This work not only provides unambiguous evidence for the crucial role of open coordination sites for the chemical adsorption of PFOA, but also represents the first demonstration of the key advantage of atomically precise engineering of adsorption sites for the highly flexible monocarboxylate PFOA using single crystal hosting structures.

## Results and Discussion

### Synthesis and Structure Analysis of PCN-999

The desymmetrized ligand 4′,4″′-(2,2-bis(4-carboxyphenyl)ethene-1,1-diyl)bis(([1,1′-biphenyl]-4-carboxylic acid)) (referred to as L12) was derived from the *D*_2*h*_-symmetric ligand 4′,4‴,4‴″,4‴‴″-(ethene-1,1,2,2-tetrayl)tetrakis(([1,1′-biphenyl]-3-carboxylic acid)) (H_4_ETTC)^33,34^ by varying the lengths of its two arms (Figure S1). Rod-like single crystals of PCN-999 suitable for SCXRD structural analysis were obtained via the solvothermal reaction of ZrCl_4_ and L12 in *N*,*N*-diethylformamide (DEF) with formic acid as a modulator (Figure S2). SCXRD study revealed that PCN-999 crystallizes in the *Cmmm* space group with lattice parameters of *a* = 27.991(2) Å, *b* = 66.287(5) Å, *c* = 29.179(2) Å, and α = β = γ = 90° (Table S1).

**Figure 1:**
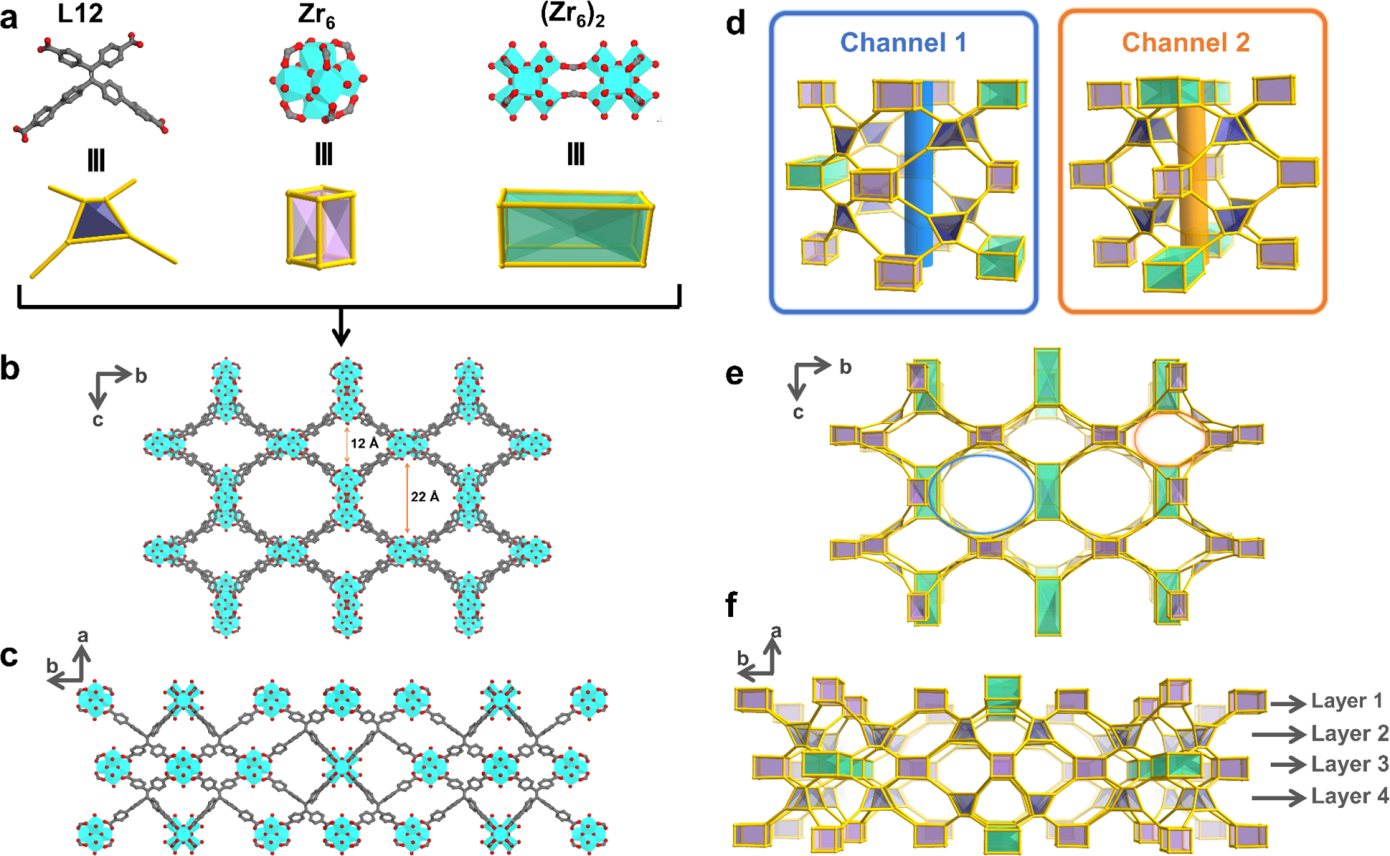
(a) Organic linker L12 and the two types of metal nodes in the extended framework. Structure illustration along the (b) *a*-axis and (c) *c*-axis. Topology illustration showing (d) two kinds of 1D channels and the packing views along the (e) *a*-axis and (f) *c*-axis. C, O, and Zr atoms are represented by gray, red, and cyan, respectively. Hydrogen atoms in the structures are omitted for clarity.

This non-interpenetrated framework exhibited two distinct metal-containing SBUs: the common [Zr_6_(μ_3_-O)_4_(μ_3_-OH)_4_(OH)_4_(H_2_O)_4_(COO)_8_] (Zr_6_) cluster and the rare {[Zr_6_(μ_3_-O)_4_(μ_3_-OH)_4_(OH)_7_(H_2_O)_7_(COO)_4_]_2_(HCOO)_2_} ((Zr_6_)_2_) SBU (Figure 1a). The (Zr_6_)_2_ SBU could be regarded as an edge-to-edge linking of two Zr_6_ clusters bridged by two formates (Figure S3). This is the first time that such a (Zr_6_)_2_ SBU was generated and utilized during the construction of MOFs. Surprisingly, both Zr_6_ and (Zr_6_)_2_ SBUs coordinate with eight crystallographically equivalent ligands (Figure S4). Interestingly, the (Zr_6_)_2_ SBUs exclusively connect to short arms, while the Zr_6_ clusters can be classified into two categories: one connecting solely to long arms (Zr_6_A) and the other linking to both long and short arms (Zr_6_B). Consequently, each L12 ligand bridges one (Zr_6_)_2_, one Zr_6_A, and two Zr_6_B clusters, yielding a novel 3D network within the (Zr_6_)_3_[(Zr_6_)_2_](L12)_8_ formulation with **scu** topology (Figure S5).

The above clusters exhibit two distinct arrangements along the *a* axis: the (Zr_6_)_2_ and Zr_6_A clusters arrange alternatively, while the Zr_6_B clusters adopt a zigzag layout (Figure S5). Such a unique arrangement gives rise to two types of 1D channels along the *a* axis with hexagonal (channel 1, ∼22 Å) and rhombic (channel 2, ∼12 Å) geometries (Figures 1b–f and S6). This structural characteristic imparts PCN-999 with a heteroporous nature, facilitating the efficient diffusion of substrates and adsorbates within its porous structure. The voids within the as-synthesized PCN-999 are occupied by disordered guest solvent molecules, constituting ∼55% of the unit cell volumes (Figure S7). Upon removal of these guest solvent molecules, PCN-999 reveals multiple open coordination sites and large accessible pores.

### Porosity and Stability Analysis

**Figure 2:**
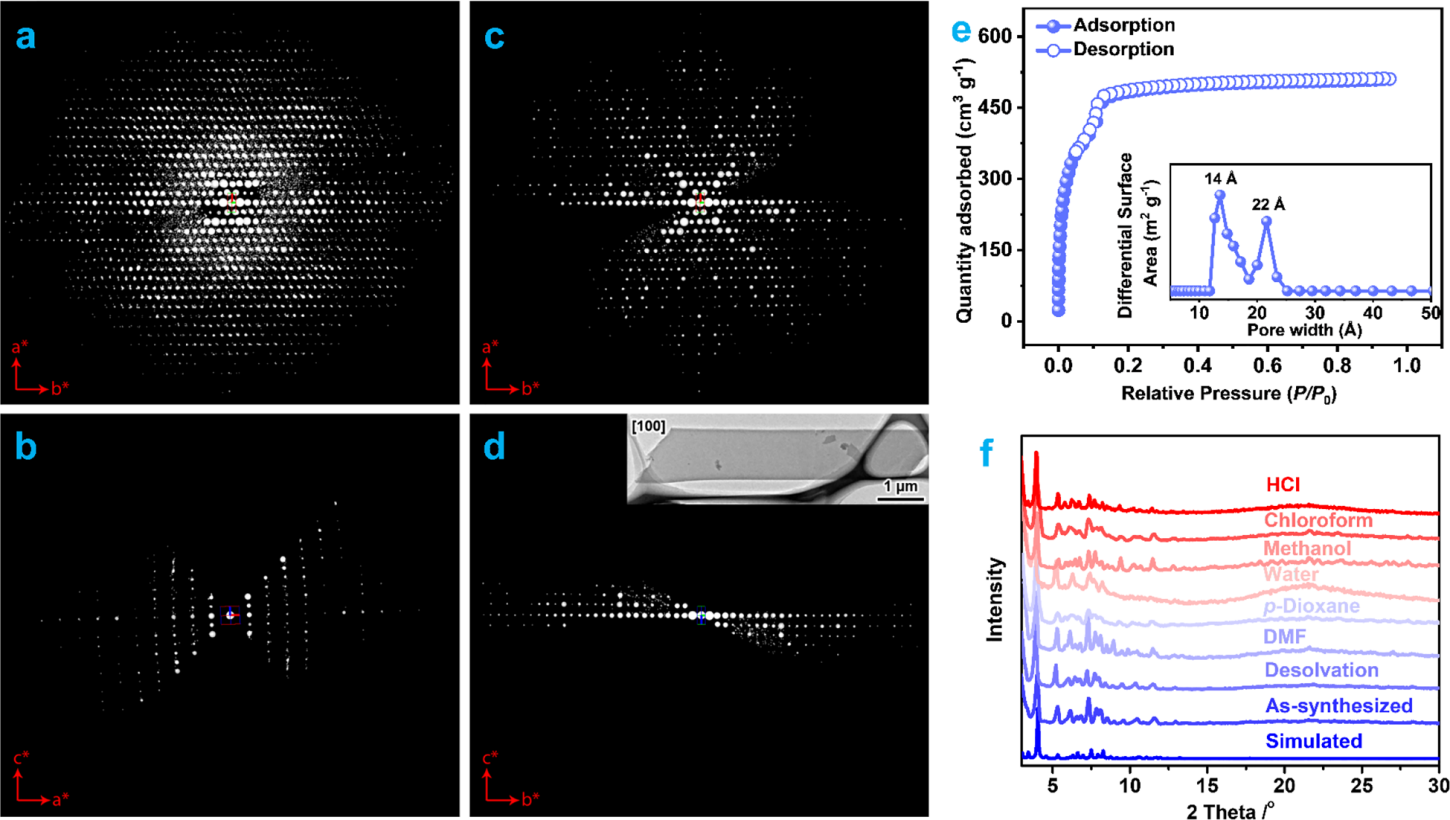
(a) Reconstructed 3D reciprocal lattice viewing along the c*-axis. 2D slice cuts from the reconstructed 3D reciprocal lattice showing the (b) h0l, (c) hk0, and (d) 0kl plane. Inset is the TEM image of PCN-999 crystal, from which the cRED data was collected. It shows that the *a*-axis is perpendicular to the nanosheet. (e) Nitrogen sorption isotherm and pore size distribution profile. (f) PXRD patterns before and after exposure to different solvents.

Powder samples were produced under slightly modified conditions from large scale synthesis. The high crystallinity of the powder sample was verified via continuous rotation electron diffraction (cRED) analysis. The 3D reciprocal lattice reconstructed from the cRED data shows that PCN-999 is crystallized in an orthorhombic system, having a *c*-centered unit cell with parameters of *a* = 28.9(2) Å, *b* = 66.2(3) Å, and *c* = 29.4(2) Å (Figure 2a–d), matching well with the SCXRD results. The porosity of PCN-999 was assessed by nitrogen adsorption–desorption measurement at 77 K. The adsorption–desorption isotherm of PCN-999 exhibits a sharp increase in the low-pressure range and a step at *ca*. 0.05–0.13 *P*/*P*_0_, indicating the presence of permanent micropores and mesopores. The Brunauer–Emmett–Teller (BET) surface area^35,36^ and Langmuir surface area of PCN-999 were calculated from the adsorption isotherm to be 1696 m^2^/g and 2237 m^2^/g, respectively (Figure S8). The total pore volume was determined to be 0.79 cm^3^/g (at *P*/*P*_0_ = 0.95), and the fitting of the isotherm by density functional theory (DFT) provides two major pore size distributions at ∼1.4 and ∼2.2 nm (Figure 2e), matching well with the hierarchical pore feature revealed in its single crystal data.

Thermogravimetric analysis (TGA) and variable-temperature powder X-ray diffraction (PXRD) measurements were conducted to test the thermal stability of PCN-999, revealing a high thermal stability with the decomposition temperature being as high as ∼450 °C and a phase transformation occurring at ∼280 °C (Figure S9). In addition, PXRD patterns show that PCN-999 retained its high crystallinity upon desolvation. The chemical stability was further evaluated by immersing the samples in different solvents, including *N,N*-dimethylformamide (DMF), *p*-dioxane, water, methanol, chloroform, and an aqueous hydrochloric acid (HCl) solution (pH = 1) for 24 h at room temperature. As shown in Figure 2f, the PXRD patterns remained identical before and after exposure to these solvents, indicating remarkable chemical stability. The high thermal and chemical stabilities can be attributed to the strong Zr–O bonds.^32^

### PFOA Uptake Study

**Figure 3:**
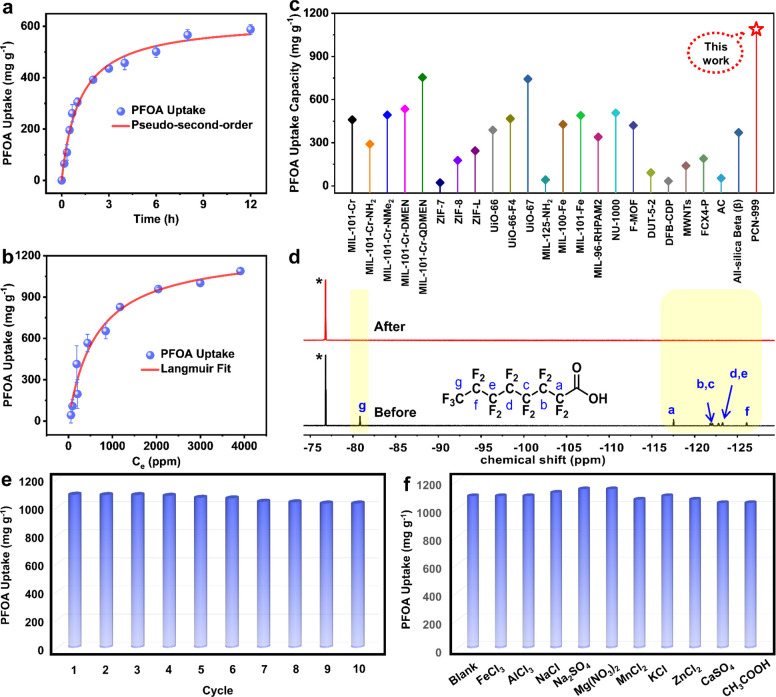
(a) Sorption kinetics of PFOA with an initial concentration of 1000 ppm, fitted with a pseudo-second-order model. (b) Equilibrium PFOA adsorption capacity as a function of equilibrium PFOA concentration (*C*_e_) fitted with the Langmuir model. (c) PFOA adsorption capacity of reported adsorbents. (d) ^19^F NMR spectra of the PFOA solution with an initial concentration of 1000 ppm before and after being treated with PCN-999. Noted: Trifluoroethanol (*) was used as an internal standard. (e) PFOA uptake after different cycles. (f) PFOA uptake in the presence of different ionic species.

The multiple open coordination sites, micromesoporous hierarchical porous structure, as well as high chemical and thermal stabilities make PCN-999 an ideal nanoscaffold for PFOA adsorption. To assess the performance of PCN-999 for the removal of PFOA from aqueous solutions, the activated PCN-999 sample was immersed into a PFOA aqueous solution with an initial concentration of 1000 ppm at room temperature. After being shaken for 3 days, the resulting PFOA-loaded MOF (PFOA@PCN-999) sample was then isolated to quantify the adsorbed PFOA. ^19^F nuclear magnetic resonance (NMR) experiments revealed a PFOA content of ∼566 mg/g within PFOA@PCN-999 (Figure S10). This outstanding performance encouraged us to further investigate the kinetics of the adsorption process (Figure S11). As shown in Figure 3a, the adsorption process exhibits fast kinetics with the equilibrium being reached within 12 h, which gives a high adsorption rate constant (*k*_2_) (1.38 × 10^–3^ g mg^–1^ h^–1^) with a high correlation coefficient (*R*^2^ = 0.99, Figure S12) by the pseudo-second order kinetics model.

The equilibrium adsorption isotherm of PFOA in PCN-999 was calculated from experimental data with initial concentrations ranging across 100–5000 ppm (Figure S13), which was then fitted by the Langmuir model (Figure 3b) with a higher correlation coefficient (0.96) than that of the Freundlich model (0.91, Figure S14). The maximum PFOA adsorption capacity was then determined to be 1089 mg/g (2.63 mmol/g) at the equilibrium concentration of 3911 ppm. To the best of our knowledge, in view of its exceptionally high PFOA uptake capacity, PCN-999 outperforms all previously reported adsorbent materials, including MOF materials^27,37^ and other adsorbents like DFB-CDP, MWNTs, FCX4-P, AC, and all-silica beta (β).^38,39^ (Figure 3c and Table S2). In addition, PCN-999 displays an extremely high removal efficiency (>99%) for a solution with an initial concentration of 1000 ppm (Figure 3d), demonstrating the high effectiveness of PCN-999 for the removal of PFOA from wastewater.

Moreover, the adsorbent PCN-999 can be easily recycled by washing with methanol (Figure S15) and then reused for subsequent PFOA adsorption cycles. Significantly, the uptake capacity remained impressively high (∼94%) even after 10 cycles (Figures 3e and S16), indicating the long cycle life of the adsorbent and further highlighting the high stability of PCN-999. In addition, the structural integrity and porosity have remained intact, as validated by the identical PXRD patterns and N_2_ sorption isotherms of PCN-999 before and after 10 cycles (Figure S17). To investigate the selective nature of PFOA adsorption in practical contexts, we immersed the activated PCN-999 sample in aqueous solutions of PFOA in the presence of different ionic species as interfering agents, including Na^+^, K^+^, Ca^2+,^ Mg^2+^, Fe^3+^, Al^3+^, Mn^2+^, Zn^2+^, Cl^–^, NO_3_^–^, SO_4_^2–^, and CH_3_COO^–^. Remarkably, the results showed identical PFOA uptake capacities across the mixtures (Figure S18), suggesting an exceptional selectivity of PCN-999 toward PFOA (Figure 3f), which can be attributed to the perfectly tailored pore size and the high affinity of PFOA for the open coordination sites. In the case of CH_3_COO^–^, the lack of interaction between the hydrophobic fluorinated carbon chains originating from the coordinated and free PFOAs will contribute to the high selectivity.

To investigate the adsorption performance of PCN-999 toward various PFAS pollutants with different chain lengths and terminal functional groups, the activated PCN-999 sample was immersed in different PFASs aqueous solutions, including perfluorobutanoic acid (PFBA), perfluorononanoic acid (PFNA), perfluorodecanoic acid (PFDA), perfluorosebacic acid (PFSEA) and perfluorobutanesulfonic acid (PFBS), with an initial concentration of 1000 ppm at room temperature (Figure S19). PCN-999 showed high adsorption capacities toward PFBA, PFNA, PFDA, and PFSEA, which is comparable to that of PFOA (Figure S20). This result suggests that variations in chain lengths do not significantly influence adsorption performance when the PFASs bear a carboxylate group. However, PCN-999 showed a poor adsorption performance toward PFBS, indicating the preference of the MOF toward carboxylate-based PFASs.

### Adsorption Mechanism Investigation

To gain in-depth insight into the PFOA binding interactions inside the MOF, further characterizations were conducted on PFOA@PCN-999. Compared to the pristine MOF, the FTIR spectrum of PFOA@PCN-999 showed the appearance of new bands at ∼1200 and ∼1140 cm^–1^ which can be ascribed to C–F stretching and the C–C–C bending vibration,^40^ respectively, indicating the successful loading of PFOA molecules. In particular, the characteristic peak corresponding to the coordinated C=O stretching mode at ∼1604 cm^–1^ shifted significantly after the adsorption of PFOA (Figure S21), suggesting interactions (Lewis acid–base interactions and coordination) between the carboxylate groups of PFOA and the unsaturated Zr sites of PCN-999. The structural integrity of PCN-999 remained after PFOA adsorption, as evidenced by the identical PXRD profiles before and after adsorption (Figure S17). The observed substantial decrease in porosity in PFOA@PCN-999 compared to pristine PCN-999, and the DFT pore size distribution calculation revealing only one major pore size at ∼1.5 nm (Figure S22), suggests the blockage of the mesopores by PFOA molecules.

**Figure 4:**
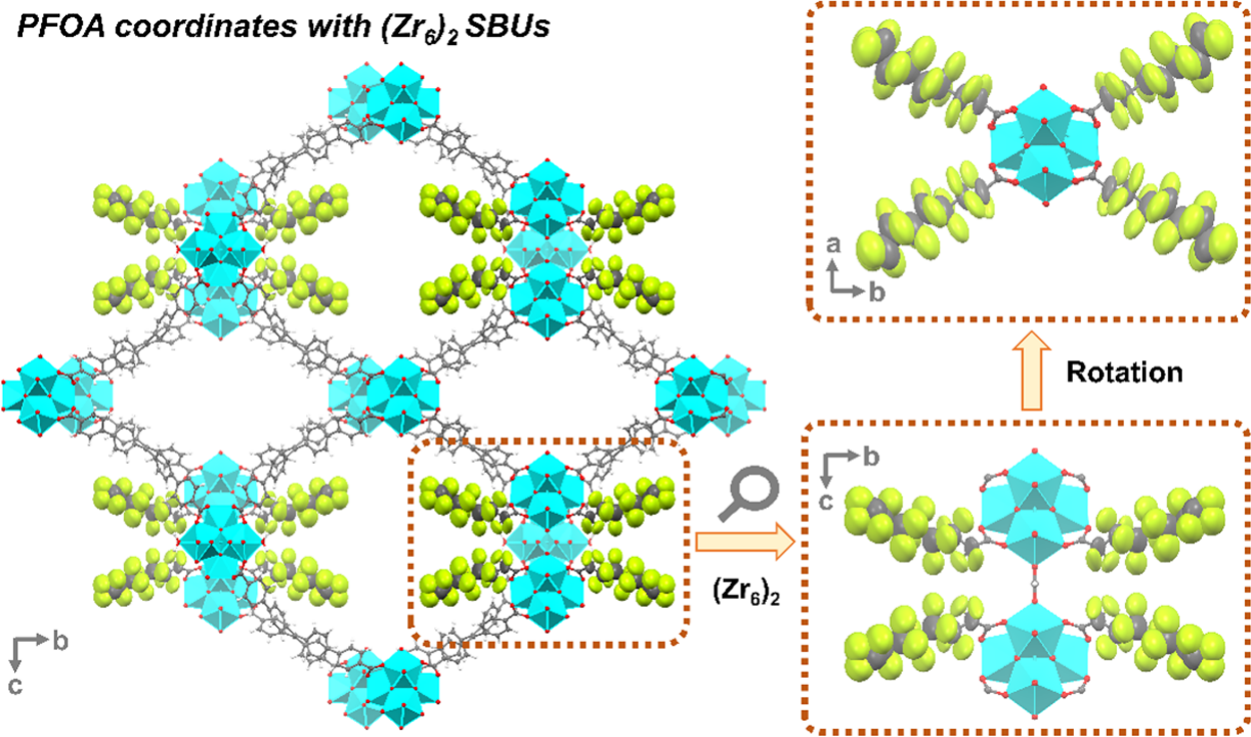
Structure of single-crystalline PFOA@PCN-999 showing the coordination of PFOA with the (Zr_6_)_2_ SBUs along the *a* axis. C, H, O, F, and Zr atoms are represented in gray, white, red, green, and cyan, respectively.

The adsorption mechanism was further explored through SCXRD analysis on PFOA@PCN-999. The as-synthesized single crystals of PCN-999 were immersed in an aqueous solution of PFOA at room temperature for 10 days. Subsequently, the single crystal data of PFOA@PCN-999 were collected at 110 K, enabling identification of the predominant location and conformation of the PFOA molecule. Compared to its pristine MOF, the space group of PFOA@PCN-999 remained *Cmmm* but with a slightly expanded lattice parameter of *a* = 28.546(2) Å, *b* = 66.369(4) Å, and *c* = 29.682(3) Å (Table S1). Crystallographically unique PFOA molecules were identified in the mesoporous channels of PCN-999 along the *a* axis (Figures 4 and S23), which exclusively coordinated with the (Zr_6_)_2_ SBUs via their carboxylate groups. This observation is in good agreement with the FTIR and pore size distribution results. Each (Zr_6_)_2_ SBU binds to eight PFOA molecules in total, forming 16 coordinated (Zr_6_)_2_ nodes. Interestingly, no PFOA was found to coordinate with the Zr_6_ clusters, underscoring the different adsorption behaviors between Zr_6_ and (Zr_6_)_2_ clusters attributed to their distinct unsaturated coordination sites.

In addition, an energetically favorable conformation, an extended “zigzag” chain, was observed for the coordinated PFOA molecule within the mesopore (Figure S24), facilitated by the ample available space. Notably, this is the first time that the coordinated PFOA molecules were directly identified by the single crystal structure, highlighting the pivotal role of additional open coordination sites in the chemical adsorption of PFOA molecules. The spatial constraints at the remaining open coordination sites hampered further coordination of PFOA. It should be noted that the much higher PFOA uptake capacity (2.63 mmol/g, equivalent to 3.14 PFOA per ligand) than the calculated value based on single crystal data (1.00 PFOA per ligand) could be assigned to the additional physical adsorption of PFOA molecules through noncovalent interactions.^3^

**Figure 5:**
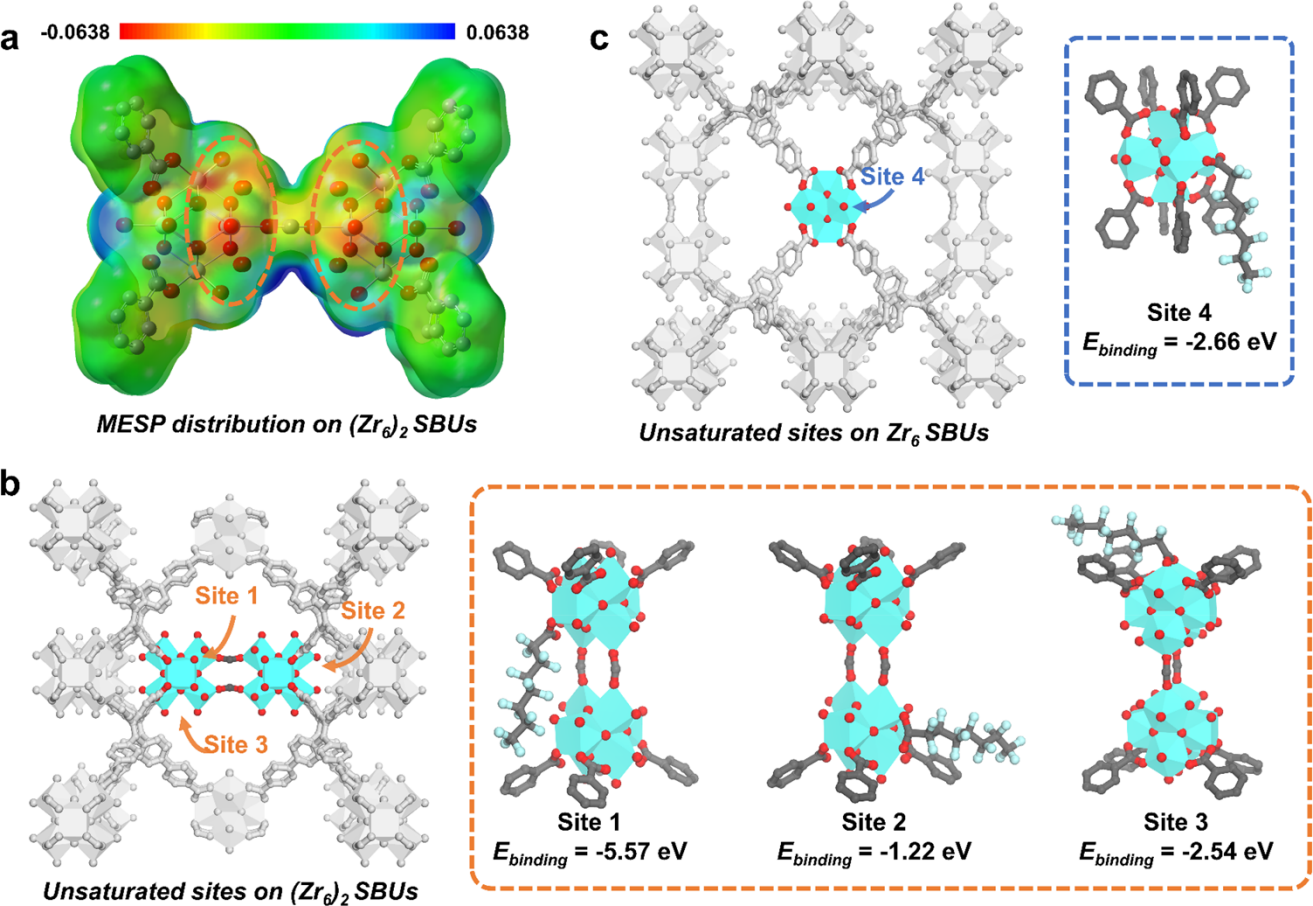
(a) Molecular electrostatic potential (MESP) distribution of the (Zr_6_)_2_ SBU. Optimized model structures of (b) (Zr_6_)_2_ and (c) Zr_6_ SBUs before and after PFOA binding on possible binding positions. C, O, F, and Zr atoms are represented by gray, red, light cyan, and cyan, respectively. Hydrogen atoms in the structures are omitted for clarity.

To cast light onto the chemical adsorption of PFOA, molecular electrostatic potential (MESP) distributions were first calculated for the two types of metal-containing SBUs. The yellow part oxygen atoms of (Zr_6_)_2_ node show negative MESP value (Figure 5a), indicating the weak coordination bond between Zr and O. In contrast, the blue part oxygen atoms of Zr_6_ cluster show positive MESP value, indicating the strong coordination bond between Zr and O (Figure S25). These results suggest that the yellow position of the (Zr_6_)_2_ SBU possesses an ability to undergo the ligand exchange process with PFOA, coinciding with the results from the molecular orbital calculation showing a slightly narrow HOMO–LUMO gap of the (Zr_6_)_2_ node (Figure S26). Furthermore, the binding energy (*E*_binding_) was calculated to get a deeper understanding of the different metal-containing SBUs. Regarding the symmetry and chemical/spatial environment of these SBUs, three potential binding sites were identified for the (Zr_6_)_2_ SBU, whereas only one feasible binding position was concerned for the Zr_6_ cluster. The cp2k combined GFN1-xtb method was applied to optimize the model structures and calculate the binding energy, which revealed that site 1 within the (Zr_6_)_2_ SBU exhibited the lowest *E*_binding_. The disparity in binding energies, with site 1 registering a value more than twice lower than that of the other sites, suggests significantly stronger adsorption at this specific site (Figure 5b,c). This result, as well as the MESP distributions, agrees well with the single crystal data, suggesting the different chemical properties in unsaturated coordination sites between (Zr_6_)_2_ and Zr_6_ clusters.

Furthermore, to deepen our understanding of the much higher experimental adsorption of PFOA beyond the single crystal data, we simulated PFOA adsorption through two distinct mechanisms: chemical bonding and physical adsorption. Positive binding energies were observed with a higher proportion of PFOA molecules forming coordination bonds (Figure S27), whereas negative binding energies were found when more PFOA molecules were physically adsorbed (Figure S28). Remarkably, a lower adsorption energy was observed upon increasing the adsorbed PFOA molecules, indicating a synergistic effect between the coordinated and free PFOA molecules due to their hydrophobicity, which can facilitate the adsorption process.^41^ The maximum adsorption capacity was calculated to be 52.58 PFOA molecules per unit cell (Figure S29), corresponding to 3.29 PFOA molecules per ligand, thereby matching the experimental adsorption capacity. Overall, these calculations further highlight the crucial role of the additional open coordination sites and a favorable porous environment in facilitating the adsorption of PFOA.

## Conclusions

In summary, we have developed a novel zirconium-based MOF equipped with Zr_6_ and unprecedented (Zr_6_)_2_ SBUs, forming a complex yet well-defined meso/microporous structured network. Significantly, each type of metal-containing SBU is intricately connected to eight ligands, resulting in the generation of multiple open coordination sites. The atomically precise structural features, coupled with its exceptional chemical and physical stability, endow PCN-999 with a remarkable PFOA adsorption performance, as quantified by an uptake capacity of 1089 mg/g (2.63 mmol/g), accompanied by rapid removal kinetics, high efficiency, and high selectivity. Our comprehensive analysis revealed the coordination of PFOA molecules exclusively with the (Zr_6_)_2_ SBU and the additional synergistic physical PFOA adsorption through the interaction between the coordinated and free PFOAs. These results highlight the power of the desymmetrization strategy in the design of MOFs with unique structures and offer unambiguous evidence for the pivotal role played by additional open coordination sites in boosting the capacity toward PFOA adsorption. These findings not only expand the structural landscape of MOFs but also improve the fundamental understanding of the MOF-based PFOA removal mechanism. Consequently, this comprehensive insight offers a significant step forward in our ability to design next-generation adsorbents for PFAS removal through the synergy of chemical and physical adsorption, establishing a critical foundation for future research in this essential area of environmental science and chemistry.

## Supporting Information Available

Experimental procedures; characterization methods; calculation methods; syntheses of L12 and PCN-999; SEM images; X-ray diffraction results; ^19^F NMR results; gas uptake analysis; thermogravimetric analysis (PDF)
