# Supplementary material for: Exceptionally High Perfluorooctanoic Acid Uptake in Water by a Zirconium-Based Metal–Organic Framework through Synergistic Chemical and Physical Adsorption
Source: J Am Chem Soc. 2024 Mar 26;146(14):9811–8. doi: 10.1021/jacs.3c14487 (PMC11009951; doi:10.1021/jacs.3c14487)
Supplement: Supplementary file 1 — ja3c14487_si_001.pdf [file ja3c14487_si_001.pdf]

# Supporting Information

## Exceptionally high perfluorooctanoic acid uptake in water by a zirconium-based metal-organic framework through synergistic chemical and physical adsorption

Rong-Ran Liang,[a,#] Shunqi Xu,[b,#] Zongsu Han,[a] Yihao Yang,[a] Kun-Yu Wang,[a] Zhehao Huang,[c] Joshua Rushlow,[a] Peiyu Cai,[a,*] Paolo Samorì,[b,*] and Hong-Cai Zhou[a,*]

[a]Department of Chemistry, Texas A&M University, College Station, TX 77843, United States.

[b]Université de Strasbourg, CNRS, ISIS, 8 alleé Gaspard Monge, 67000 Strasbourg, France.

[c]Department of Materials and Environmental Chemistry, Stockholm University, SE-106 91 Stockholm, Sweden.

Contents

Section 1. General methods and instruments ....3

Section 2. Ligand and MOF Synthesis ....5

Section 3. Characterizations of the MOFs ....6

Figure S1. Design of the desymmetrized ligand L12 ....6

Figure S2. SEM images of PCN-999. ....6

Figure S3. The geometry of L12 and the unique $(Zr_6)_2$ SBU in PCN-999. ....6

Figure S4. Demonstration of the coordination of the ligand with $Zr_6$ cluster and $(Zr_6)_2$ SBU in PCN-999. ....7

Figure S5. The connection between L12 and SBUs, the alternative arrangement of $(Zr_6)_2$ and $Zr_6$A SBUs, the zig-zag layout of $Zr_6$B clusters, and the scu topology of PCN-999 ....7

Figure S6. Packing structure of PCN-999 ....7

Figure S7. Demonstration of the solvent accessible void of PCN-999. ....8

Figure S8. The consistency plot, BET surface area plot, and Langmuir surface area plot of PCN-999. ....8

Figure S9. TGA profile and the variable-temperature PXRD patterns of PCN-999. ....8

Figure S10. $^{19}F$ NMR spectrum of the digested PCN-999 after immersing in 1000 ppm PFOA solution. ....9

Figure S11. $^{19}F$ NMR spectra of the digested PCN-999 samples after immersing in 1000 ppm PFOA solution for different time ....9

Figure S12. Sorption kinetics of PFOA into PCN-999 ....10

Figure S13. $^{19}F$ NMR spectra of the digested PCN-999 samples after immersing in different

PFOA solutions........................................................................................................10
Figure S14. Equilibrium PFOA adsorption capacity of PCN-999 ..............................................10
Figure S15. $^{19}$F NMR spectrum of the digested PCN-999 sample after PFOA desorption.........11
Figure S16. $^{19}$F NMR spectra of the digested PCN-999 samples after different cycles.............11
Figure S17. PXRD patterns of PCN-999 and $N_2$ adsorption-desorption isotherms of PCN-999 before and after 10 cycles. ..........................................................................................11
Figure S18. $^{19}$F NMR spectra of the digested PCN-999 samples after immersing in different PFOA solutions with the presence of different ions ..................................................12
Figure S19. $^{19}$F NMR spectra of the digested PCN-999 samples after immersing in different PFAS solutions........................................................................................................12
Figure S20. PFAS uptake of PCN-999 in 1000 ppm aqueous solutions. ..................................12
Figure S21. FTIR spectra of $ZrCl_4$, L12, PCN-999, PFOA@PCN-999, and PFOA...................13
Figure S22. $N_2$ sorption isotherm of PFOA@PCN-999 ..........................................................13
Figure S23. Packing structure of PFOA@PCN-999....................................................................13
Figure S24. PFOA coordination on the $(Zr_6)_2$ SBU .................................................................14
Figure S25. Molecular electrostatic potential (MESP) distributions .........................................14
Figure S26. Molecular orbital calculation of $(Zr_6)_2$ and $Zr_6$ SBUs............................................15
Figure S27. Simulated adsorption of more PFOA molecules through chemical bonding within PCN-999. ........................................................................................................................15
Figure S28. Simulated adsorption of more PFOA molecules through physical adsorption within PCN-999. ........................................................................................................................15
Figure S29. Simulated PFOA adsorption isotherm of PCN-999 in ambient condition..............16
Figure S30. $^{1}$H NMR spectrum of L12. ....................................................................................16
Figure S31. $^{13}$C NMR spectrum of L12. ...................................................................................17
Figure S32. Mass spectrum of L12. ..........................................................................................17
Table S1. Crystallographic data and structural refinement summary. ......................................17
Table S2. PFOA adsorption capacity of different reported adsorbents......................................18
Reference ...................................................................................................................................19

## Section 1. General methods and instruments

**Nuclear Magnetic Resonance (NMR) spectroscopy.** $^{1}H$ NMR, $^{13}C$ NMR, and $^{19}F$ NMR spectra were obtained on a AVANCE NEO 400 spectrometer.

**Powder X-ray diffraction (PXRD).** PXRD patterns were collected on a Bruker D8 Advance ECO powder diffractometer with a Cu microfocus tube ($\lambda$ = 1.54178 Å) at 40 kV and 25 mA.

**Thermogravimetric analysis (TGA)**

TGA measurement was conducted on a Mettler-Toledo TGA/DSC 1 under nitrogen ($N_2$) atmosphere with a ramp rate of 5 °C/min from room temperature and 800 °C.

**Scanning electron microscopy (SEM)**

SEM was carried out using a FEI QUANTA 600 FE-SEM scanning electron microscope. The samples were dispersed over the slices of silicon wafer adhered to flat copper platform sample holders and then coated with gold using a sputter coater (ambient temperature, 85 torr pressure in a nitrogen atmosphere, puttered for 30s from a solid gold target at a current at 30 mA) before being submitted to SEM characterization.

**Fourier transform infrared spectroscopy (FTIR)**

FTIR data were collected on a SHIMADZU IRAffinity-1 FTIR Spectrophotometer.

**Nitrogen ($N_2$) sorption measurement.** $N_2$ adsorption-desorption measurement was performed on a Micromeritics ASAP 2020 system. Prior to the measurement, the as-synthesized PCN-999 sample was washed with N,N-dimethylformamide (DMF) to remove the unreacted starting materials, followed by the exchange with acetone for several times to remove the non-volatile DMF. The resulting sample was then activated under vacuum at 80 °C for 12 h. The $N_2$ adsorption-desorption isotherm was then measured at 77 K, from which the specific surface area was generated using the Brunauer-Emmett-Teller (BET) model.

**Single-crystal X-ray Crystallography.**

The single crystals of PCN-999 before and after perfluorooctanoic acid (PFOA) loading were directly transferred from the mother liquid to the oil, and then mounted onto a loop for single crystal X-ray diffraction measurements (SCXRD). The data were collected on a Bruker D8-Venture diffractometers equipped with Cu microfocus tubes ($\lambda$ = 1.54178 Å) and low temperature device. The single crystal structures were solved and refined using Olex2 software.[1] Both structures were solved by the direct method using the *SHELXT* program and refined by full-matrix least-squares method with *SHELXL* package.[2] All non-hydrogen atoms were refined with anisotropic displacement parameters, and the hydrogen atoms were positioned by geometrical calculation and then refined by riding. The free solvent molecules are highly disordered in MOFs and attempts to locate and refine the solvent peaks were unsuccessful. The diffused electron densities resulting from these solvent molecules were removed using the solvent MASK. Crystal data are summarized in Table S1 and the single crystal structures can be obtained free of charge from The Cambridge Crystallographic Data Centre with the CCDC number of 2304331 and 2304332.

**Calculation method.**

The cp2k combined GFN1-xtb method was applied to optimize the model structures and calculate the binding energy ($E_{binding}$) using the equation of $E_{binding} = E_{MOF+PFOA} - (E_{MOF} + E_{PFOA})$, where $E_{MOF+PFOA}$ is the total energy of the MOF model compound binding with PFOA, $E_{PFOA}$ is the total energy of *PFOA*, and $E_{MOF}$ is the sum of the total energy of the MOF model compound. The more negative the value of $E_{binding}$, the stronger the PFOA adsorption. Simulated PFOA adsorption isotherm was generated using the sorption module in Materials Studio 2020 software.

**PFASs adsorption from aqueous solutions.**

Prior to the PFASs adsorption experiments, the as-synthesized PCN-999 sample was washed with DMF and acetone several times, followed by vacuuming at 80 $^{o}$C overnight. PFASs adsorption experiment was performed in aqueous solutions with a ratio of m/V = 1 (1 mg of MOF in 1 mL of solution). After shaking for certain time at room temperature, the supernatant was decanted, and the PFAS-loaded MOF (PFAS@PCN-999) sample was washed with water and dried. Subsequently, the PFAS@PCN-999 samples were fully digested using a mixture of DMSO-$d_6$ and deuterated sulfuric acid (50/3, v/v) by heating at 80 $^{o}$C for 6 h. 4-Trifluoromethyl benzoic acid was used as the internal standard for the quantification of adsorbed PFAS inside the MOF by integrating the area of the PFAS signal and the internal standard signal, respectively.

**PFOA removal efficiency experiment.**

PFOA removal efficiency experiment was performed by adding PCN-999 (3 mg) into the aqueous PFOA solution (1000 ppm, 0.6 mL, deuterium oxide). After shaking for 1 day at room temperature, the supernatant was detected by $^{19}$F NMR using trifluoroethanol as the internal standard.

**MOF regeneration and recycling experiment.**

PCN-999 was regenerated from PFOA@PCN-999 samples by immersing in methanol for ~30 h at room temperature. The recycling experiment was conducted by repeating the PFOA adsorption and desorption experiments successively.

**Selective adsorption of PFOA over different ionic species.**

Selective PFOA adsorption experiment was performed in aqueous solutions with a ratio of m/V = 1 (1 mg of MOF in 1 mL of solution). The concentration of different species in the aqueous PFOA solutions is 250 ppm. After shaking for 3 days at room temperature, the supernatant was decanted, and the guest-loaded PCN-999 sample was washed with water and dried. Subsequently, the guest-loaded PCN-999 samples were fully digested using a mixture of DMSO-$d_6$ and deuterated sulfuric acid (50/3, v/v) by heating at 80 $^{o}$C for 6 h. 4-Trifluoromethyl benzoic acid was used as the internal standard for the quantification of adsorbed PFOA inside the MOF.

**Section 2. Ligand and** MOF Synthesis

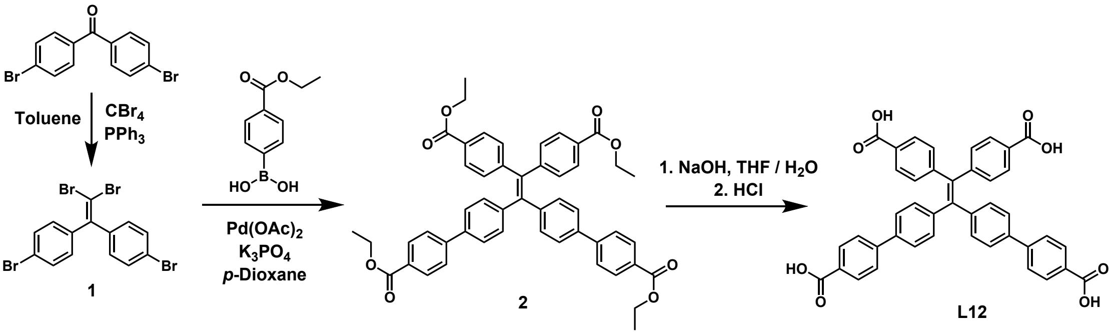


*Synthesis of 4,4'-(2,2-dibromoethene-1,1-diyl)bis(bromobenzene) (**1**).* To a solution of bis(4-bromophenyl)methanone (1.00 g, 2.94 mmol) in anhydrous toluene (50 mL) was added carbon tetrabromide ($CBr_4$, 2.00 g, 6.03 mmol) and triphenylphosphine (2.20 g, 8.39 mmol), and the mixture was heated at 140 $^oC$ for 3 days. After cooling to room temperature, the precipitate was filtered and washed with toluene, and the filtrate was collected and washed with water and brine. The organic layer was separated and dried with $MgSO_4$, and then the solvent was removed under reduced pressure. The resulting residue was purified by column chromatography on silica gel using hexane/dichloromethane as the eluent to give compound **1** as light-yellow solid (0.52 g, Yield 36%)

*Synthesis of diethyl 4',4'''-(2,2-bis(4-(ethoxycarbonyl)phenyl)ethene-1,1-diyl)bis([1,1'-biphenyl]-4-carboxylate)* (**2**). Compound **1** (2.40 g, 4.84 mmol), (4-(ethoxycarbonyl)phenyl)boronic acid (5.80 g, 29.90 mmol), $K_3PO_4$ (8.22 g, 38.72 mmol), and Palladium(II) acetate (0.06 g, 0.27 mmol) were added to a 500-mL Schlenk flask charged with a stir bar. The flask was pumped under vacuum and refilled with $N_2$ three times before 250 mL degassed 1,4-dioxane was transferred to the system. The reaction mixture was refluxed for 72 h under a $N_2$ atmosphere. After the reaction mixture cooled to room temperature, the organic solvent was removed using a rotary evaporator, and the resulting mixture was poured into water and extracted with dichloromethane (3 × 100 mL). The combined organic layers were dried over anhydrous $MgSO_4$, and then the solvent was removed under reduced pressure. After purification by column chromatography on silica gel using dichloromethane/hexane as eluent, compound **2** was obtained as yellow solid (2.09 g, yield: 56 %).

*Synthesis of 4',4'''-(2,2-bis(4-carboxyphenyl)ethene-1,1-diyl)bis(([1,1'-biphenyl]-4-carboxylic acid))* (**L12**). Compound **2** (1.00 g, 2 mmol) was dissolved in 40 mL of THF, to which 40 mL of 10 M NaOH aqueous solution was added. The mixture was stirred under reflux overnight, and then the organic solvent was removed under reduced pressure. The aqueous phase was acidified to pH = 1 using concentrated HCl aqueous solution. The resulting precipitate was collected via filtration, washed with water, and dried under vacuum to afford **L12** (0.73 g, 85 %). $^1H$ NMR (400 MHz, DMSO-$d_6$) δ 12.94 (s, 4H), 7.97 (d, $J$ = 8.5 Hz, 4H), 7.76 (dd, $J$ = 8.5, 4.6 Hz, 8H), 7.60 (d, $J$ = 8.5 Hz, 4H), 7.15 (dd, $J$ = 10.7, 8.3 Hz, 8H). $^{13}C$ NMR (100 MHz, DMSO-$d_6$) δ 167.55, 167.44, 147.68, 143.78, 142.81, 142.28, 140.16, 137.89, 132.02, 131.41, 130.41, 130.12, 129.62, 127.06, 126.97. HRMS (-ESI): Calcd. $[M-H]^-$ *m/z*: 659.1700. Found: 659.1713.

**Synthesis of PCN-999**.

L12 (5 mg), $ZrCl_4$ (10 mg), formic acid (800 μL), and diethylformamide (DEF) (2/1.5 mL) were charged in a 4 mL Pyrex vial. After sonication of around 10 minutes, the mixture was then heated at 120 °C for 10 days. After cooling to room temperature, the flaky single crystals of PCN-999 were harvested. The

powder sample of PCN-999 was obtained by mixing L12 (20 mg), $ZrCl_4$ (40 mg), formic acid (1.2 mL), and DEF (3 mL) in a 20 mL vial, followed by heating at 120 °C for 3 days to give the yellow powder (yield: 71 %).

**Section 3. Characterizations of the MOFs**

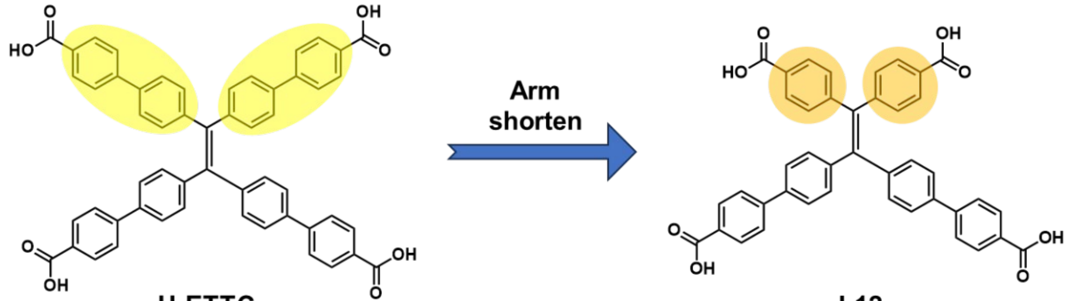


$H_4ETTC$ L12

**Figure S1.** Design of the desymmetrized ligand L12 through the derivation from the $D_2$h-symmetric ligand ($H_4ETTC$) by changing the length of its two arms.

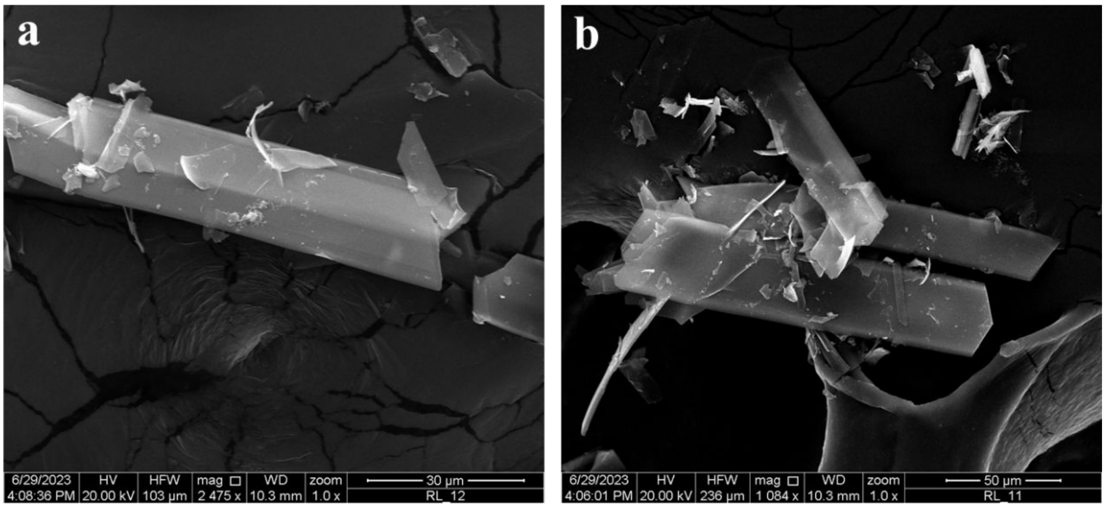


**Figure S2.** SEM images of PCN-999.

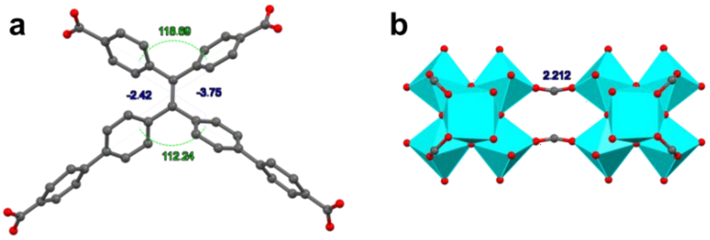


**Figure S3.** (a) The geometry of L12 in PCN-999. (b) The unique $(Zr_6)_2$ SBU in PCN-999.

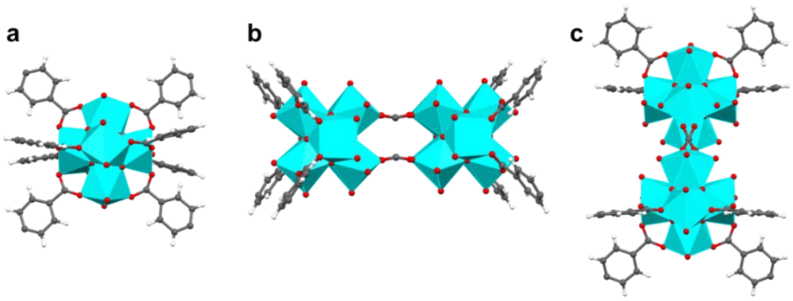


**Figure S4.** Demonstration of the coordination of the ligand with (a) $Zr_6$ cluster, and (b,c) $(Zr_6)_2$ SBU in PCN-999.

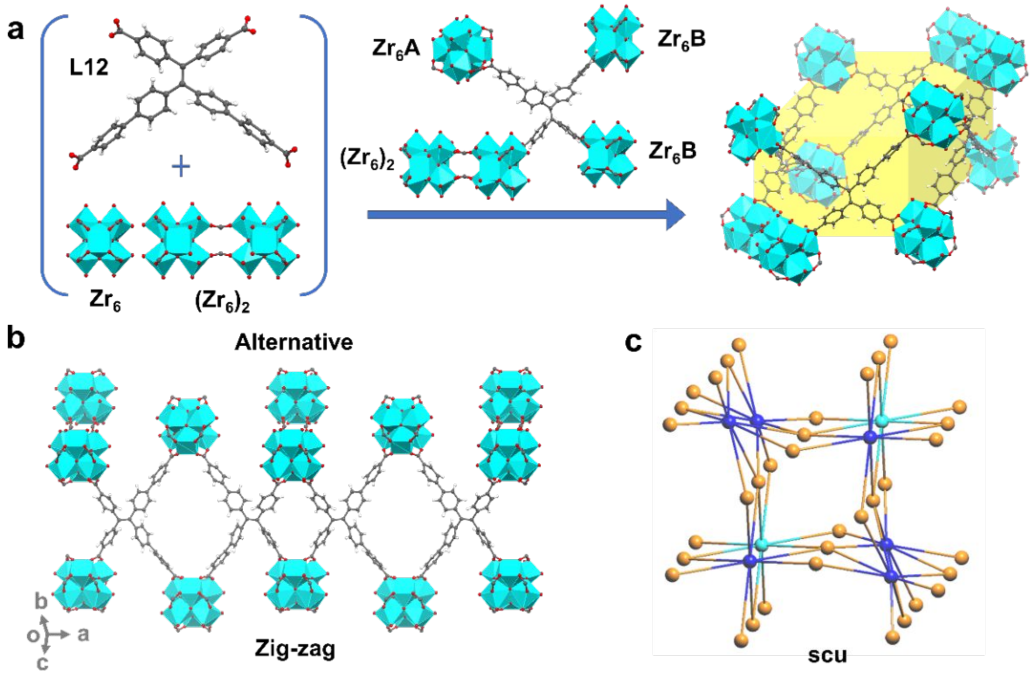


**Figure S5.** (a) The connection between L12 and two kinds of SBUs, $Zr_6$ and $(Zr_6)_2$, to form PCN-999 network. (b) The alternative arrangement of $(Zr_6)_2$ and $Zr_6$A SBUs, and the zig-zag layout of $Zr_6$B clusters. (c) The **scu** topology of PCN-999. C, H, O, and Zr atoms are represented by gray, white, red, and cyan, respectively.

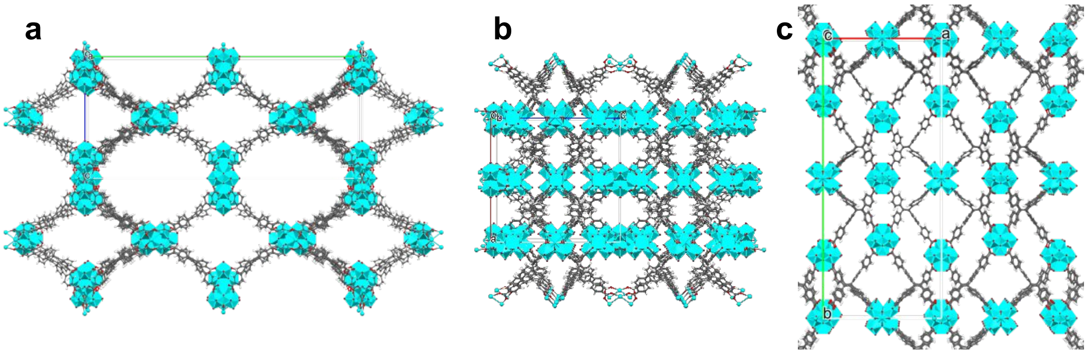


**Figure S6.** Packing structure of PCN-999 along (a) *a* axis, (b) *b* axis, and (c) *c* axis.

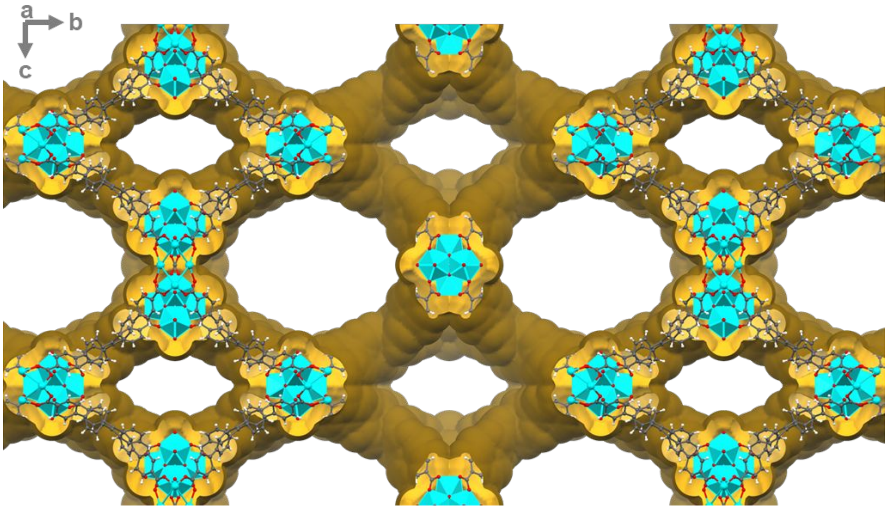


**Figure S7.** Demonstration of the solvent accessible void of PCN-999.

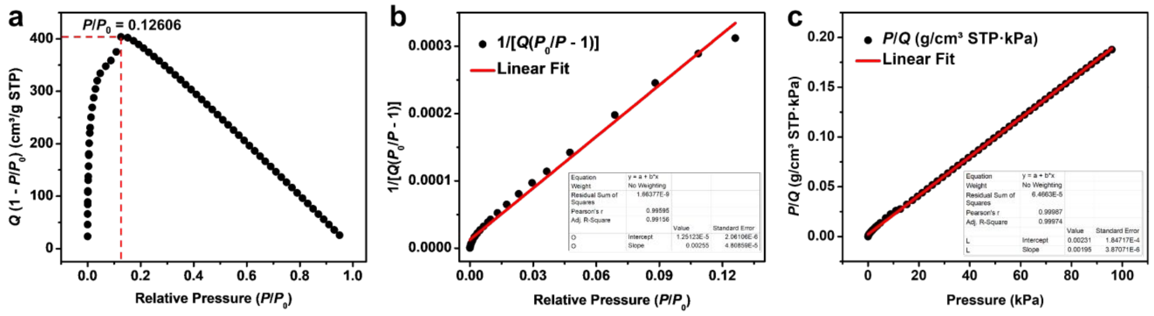


**BET constant** $C = 1 + 0.00255 / (1.25123 \times 10^{-5}) = 205$

$$(P/P_0)_{n_m} = \frac{1}{\sqrt{C}+1} = 0.06528$$

**Figure S8.** (a) The consistency plot, (b) BET surface area plot, and (c) Langmuir surface area plot of PCN-999.

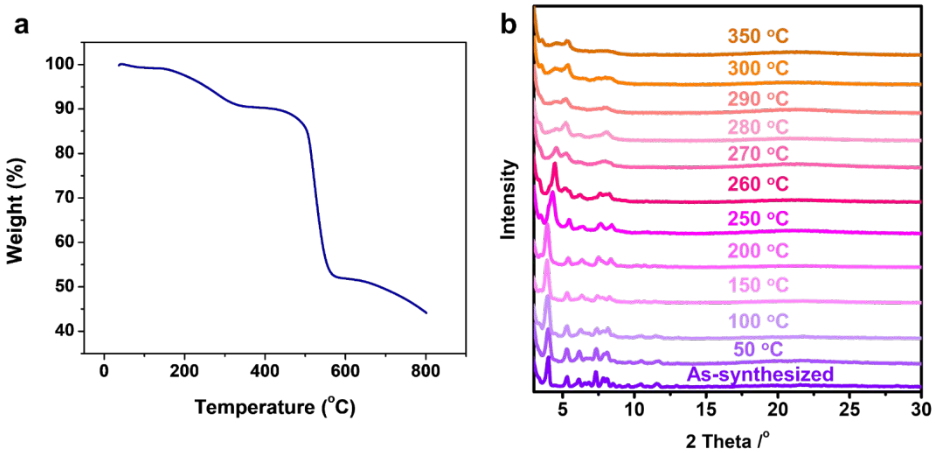


**Figure S9.** (a) TGA profile and (b) the variable-temperature PXRD patterns of PCN-999.

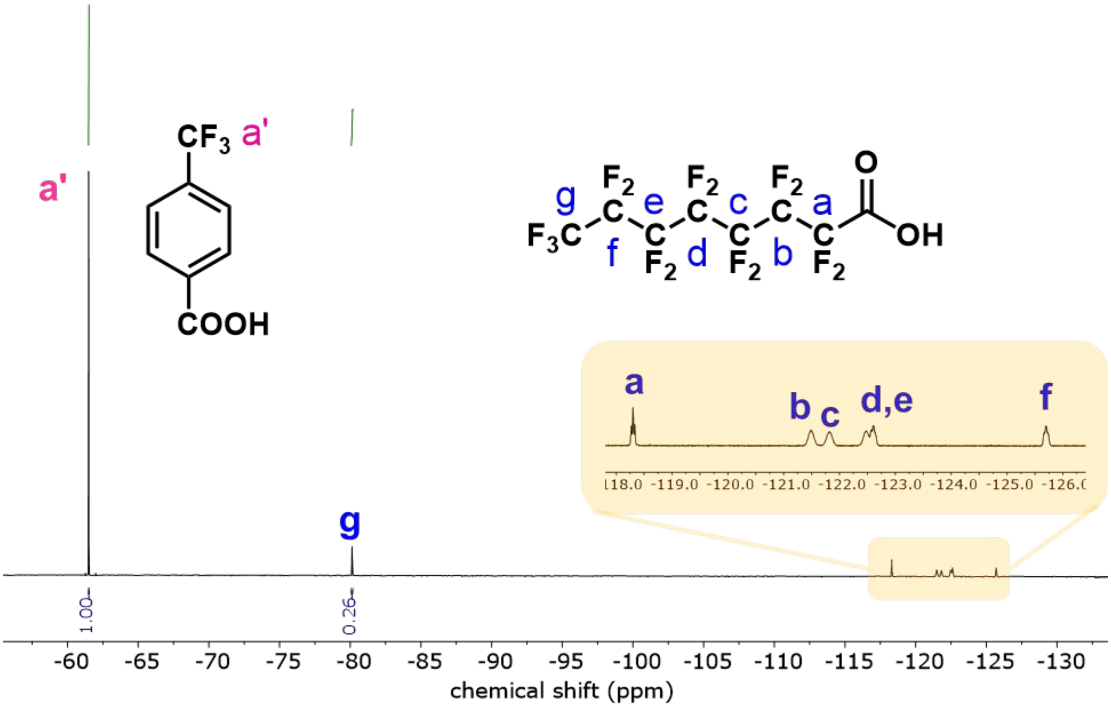


**Figure S10.** $^{19}$F NMR spectrum of the digested PCN-999 after immersing in 1000 ppm PFOA solution for 3 days. Noted: 4-(Trifluoromethyl)benzoic acid was used as an internal standard.

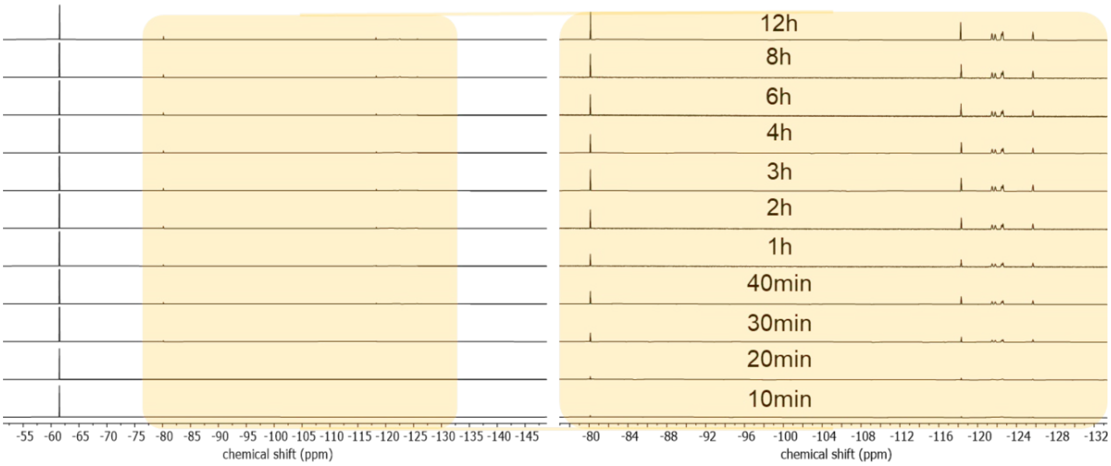


**Figure S11.** $^{19}$F NMR spectra of the digested PCN-999 samples after immersing in 1000 ppm PFOA solution for different time. Noted: 4-(Trifluoromethyl)benzoic acid was used as an internal standard.

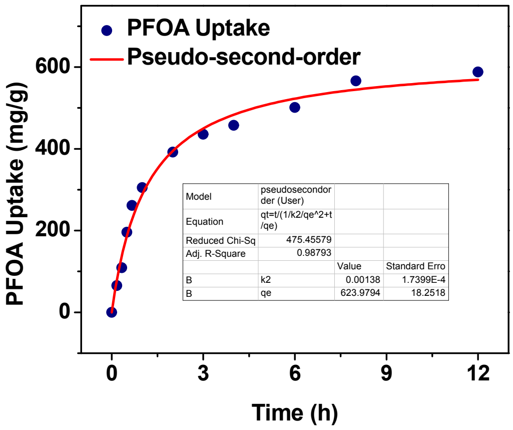


**Figure S12.** Sorption kinetics of PFOA into PCN-999 with an initial concentration of 1000 ppm, fitted with a pseudo-second-order model.

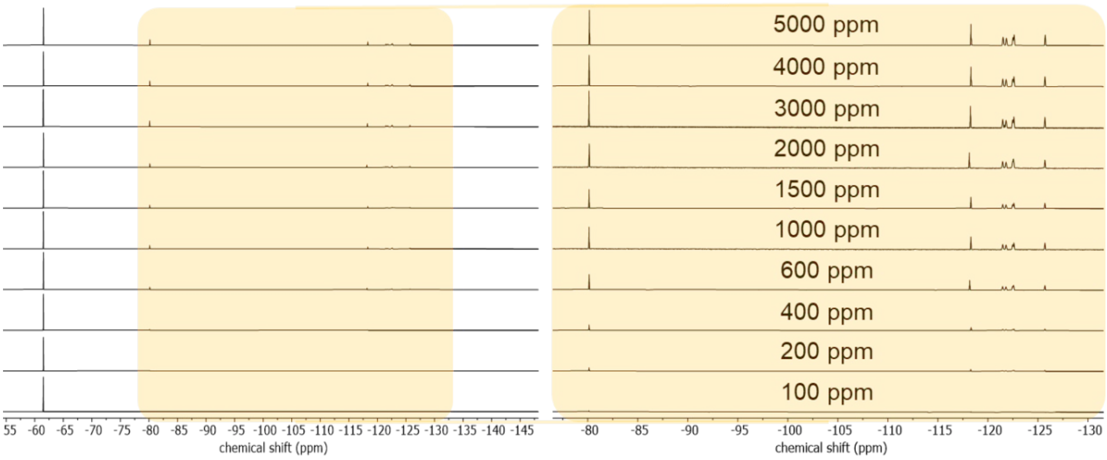


**Figure S13.** $^{19}$F NMR spectra of the digested PCN-999 samples after immersing in different PFOA solutions for 3 days. Noted: 4-(Trifluoromethyl)benzoic acid was used as an internal standard.

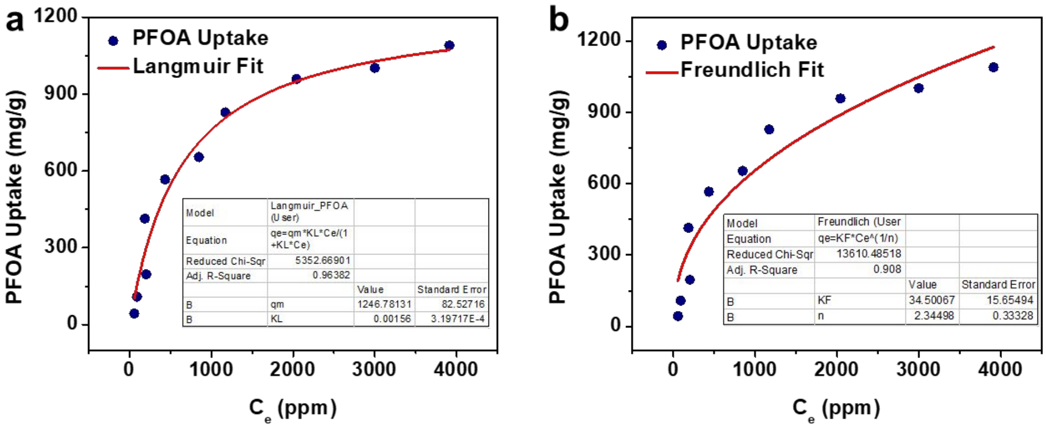


**Figure S14.** Equilibrium PFOA adsorption capacity of PCN-999 as a function of equilibrium PFOA concentration ($C_e$) fitted with (a) Langmuir model and (b) Freundlich model.

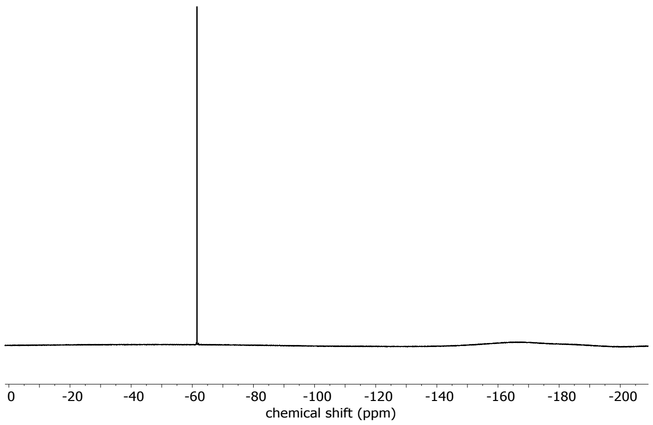


**Figure S15.** $^{19}F$ NMR spectrum of the digested PCN-999 sample after PFOA desorption. Noted: 4-(Trifluoromethyl)benzoic acid was used as an internal standard.

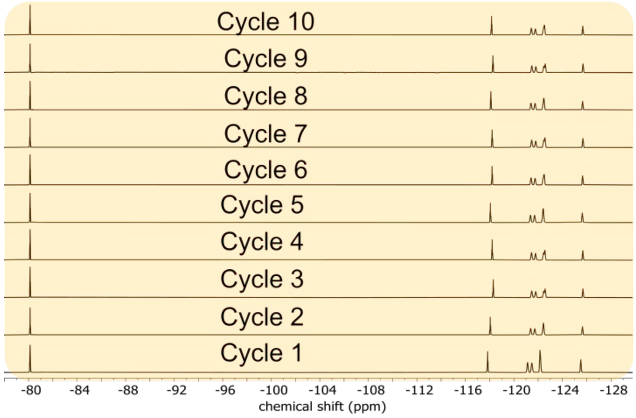


**Figure S16.** $^{19}F$ NMR spectra of the digested PCN-999 samples after immersing in 5000 ppm PFOA solution for different cycles. Noted: 4-(Trifluoromethyl)benzoic acid was used as an internal standard.

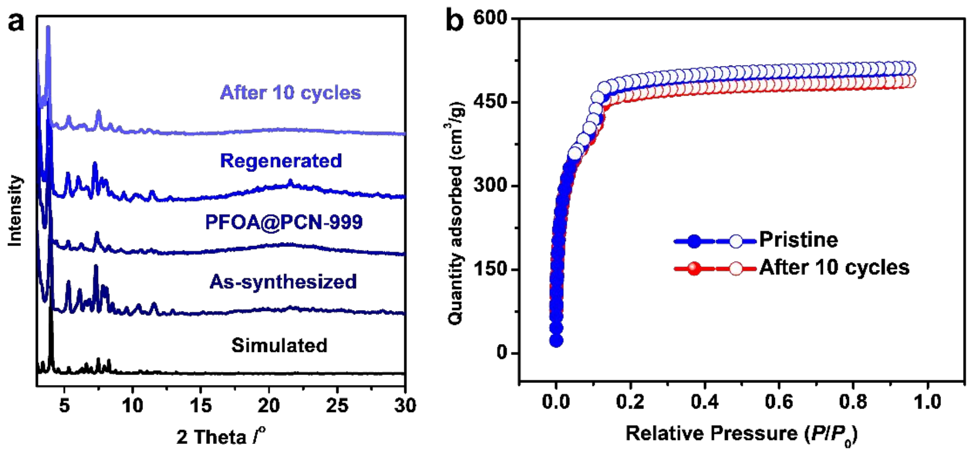


**Figure S17**. (a) PXRD patterns of PCN-999. (b) $N_2$ adsorption-desorption isotherms (77K) of PCN-999 before and after 10 cycles.

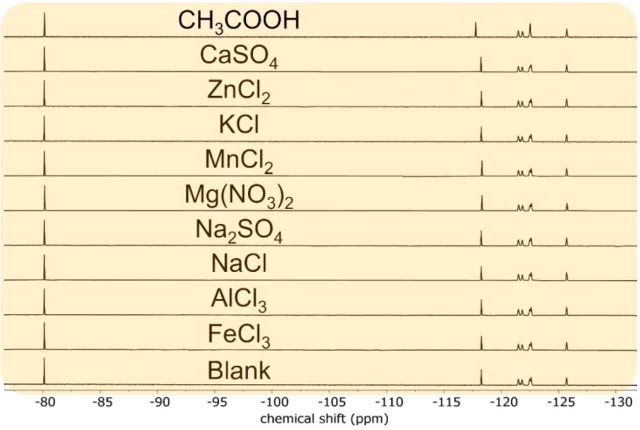


**Figure S18.** $^{19}$F NMR spectra of the digested PCN-999 samples after immersing in different PFOA solutions with the presence of different ions for 3 days. Noted: 4-(Trifluoromethyl)benzoic acid was used as an internal standard.

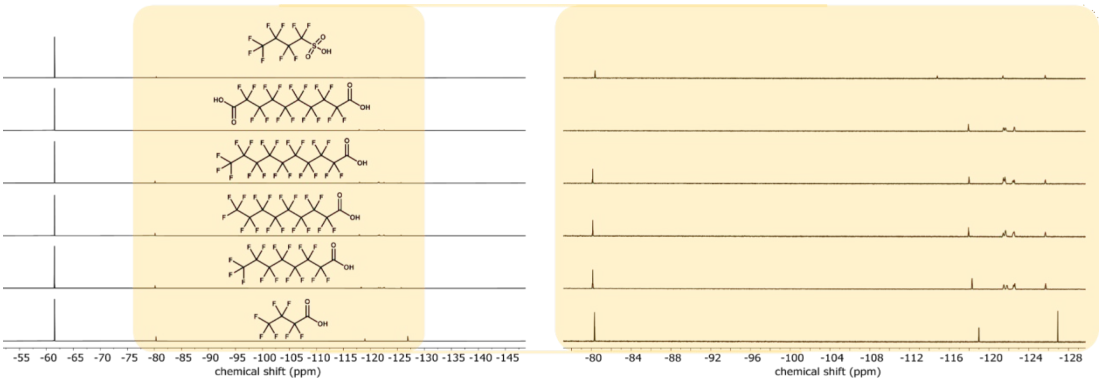


**Figure S19.** $^{19}$F NMR spectra of the digested PCN-999 samples after immersing in different PFAS solutions with an initial concentration of 1000 ppm. Noted: 4-(Trifluoromethyl)benzoic acid was used as an internal standard.

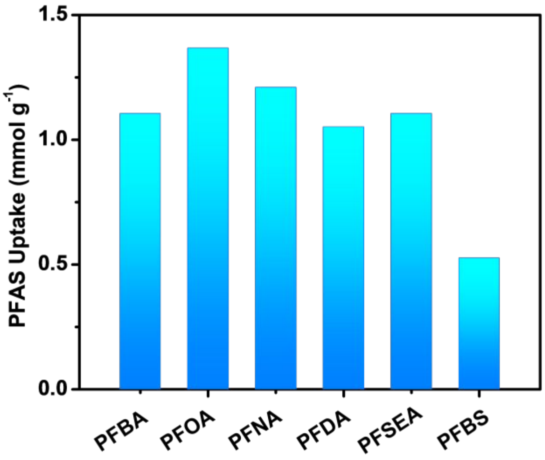


**Figure S20.** PFAS uptake of PCN-999 in 1000 ppm aqueous solutions.

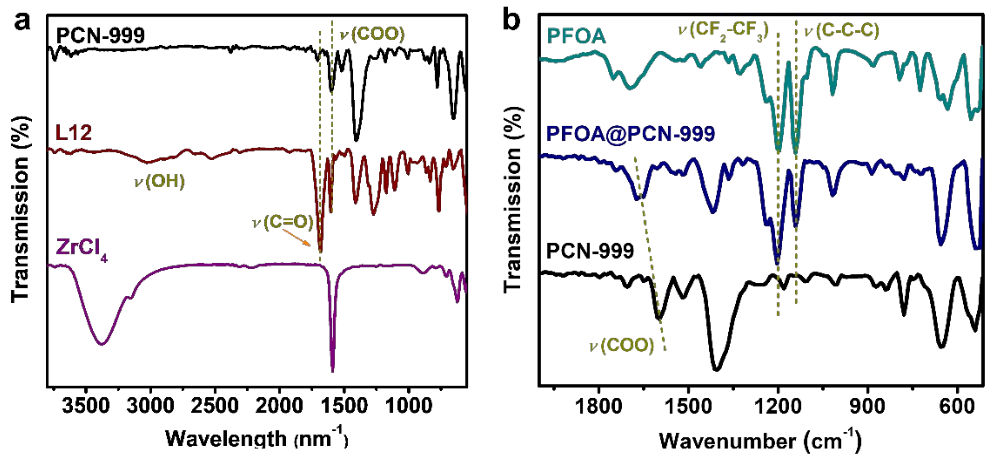


**Figure S21.** FTIR spectra of $ZrCl_4$, L12, PCN-999, PFOA@PCN-999, and PFOA.

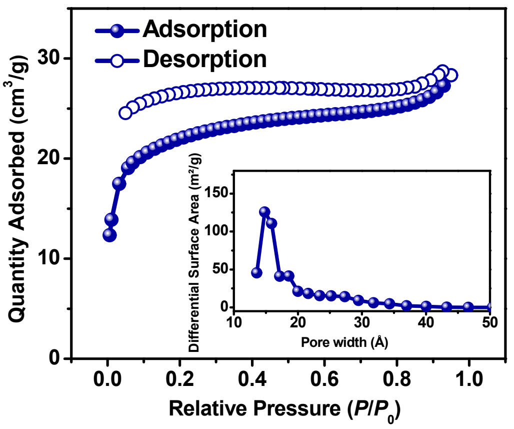


**Figure S22.** $N_2$ sorption isotherm of PFOA@PCN-999 with the pore size distribution profile inserted.

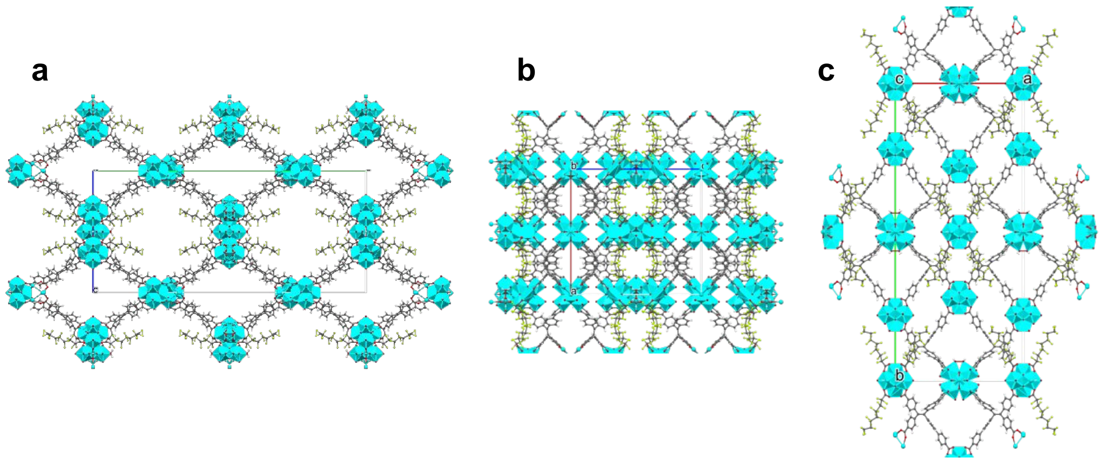


**Figure S23.** Packing structure of PFOA@PCN-999 along (a) *a* axis, (b) *b* axis, and (c) *c* axis.

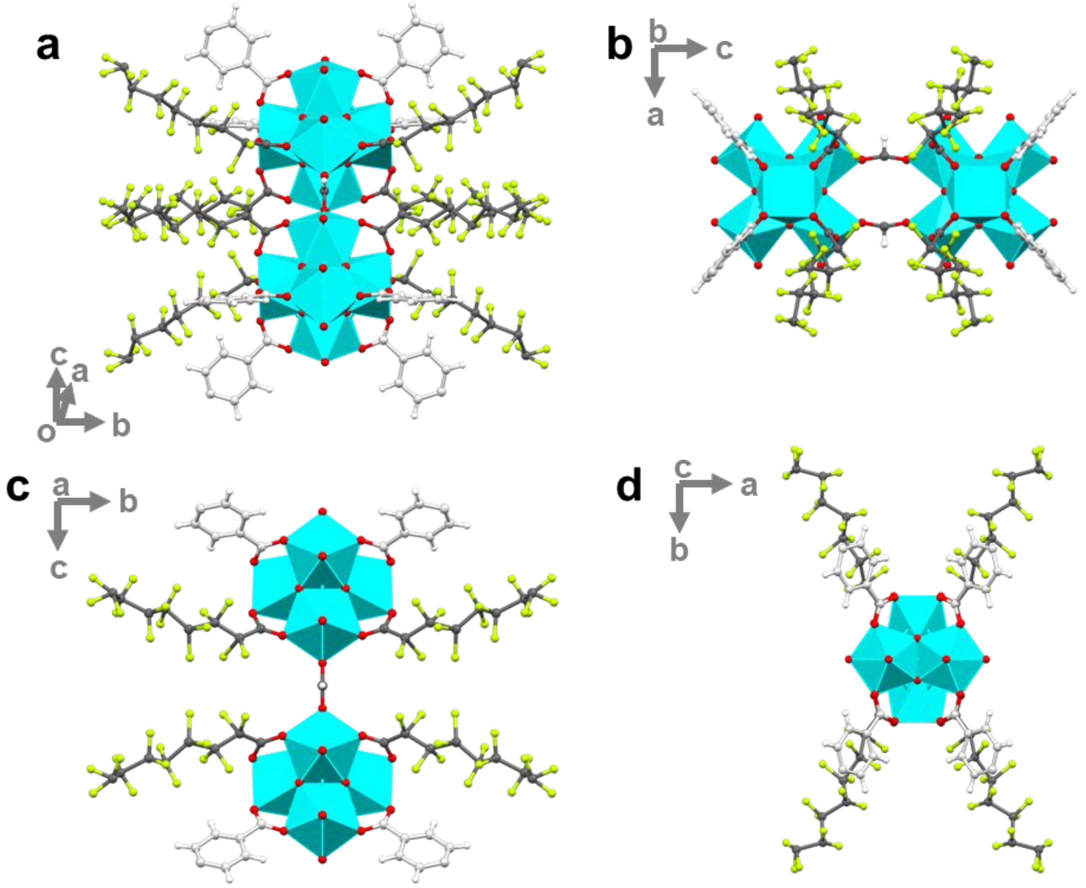


**Figure S24.** PFOA coordination on the $(Zr_6)_2$ SBU showed along (a) *bc* face, (b) *b* axis, (c) *a* axis, and (d) *c* axis.

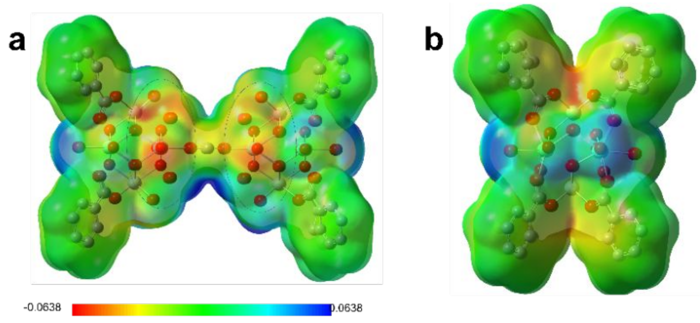


**Figure S25.** Molecular electrostatic potential (MESP) distributions of (a) $(Zr_6)_2$ and (b) $Zr_6$ SBUs. Blue region represents the positive MESP value (e.g., nucleophilic centers), while the red region stands for the negative MESP value (e.g., electrophilic center). Protons in the structures are removed for clarity.

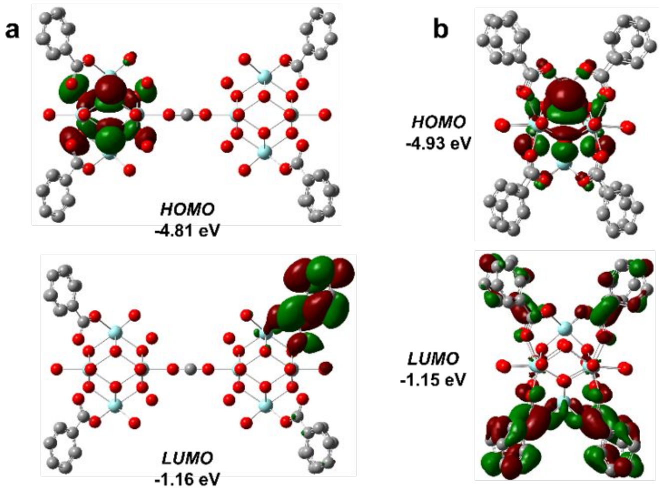


**Figure S26.** Molecular orbital calculation of (a) $(Zr_6)_2$ and (b) $Zr_6$ SBUs. Protons in the structures are removed for clarity.

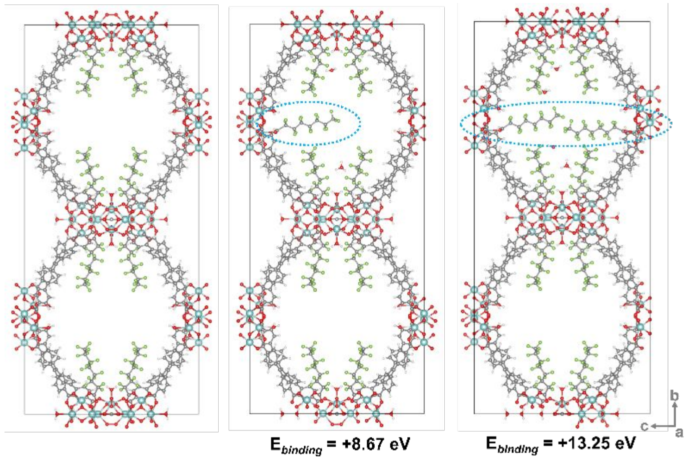


**Figure S27.** Simulated adsorption of more PFOA molecules through chemical bonding within PCN-999.

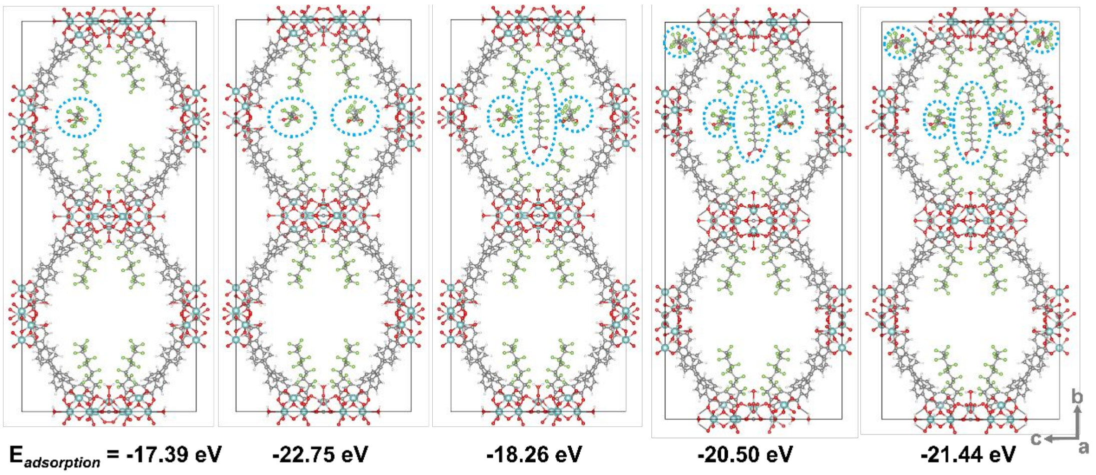


**Figure S28.** Simulated adsorption of more PFOA molecules through physical adsorption within PCN-999.

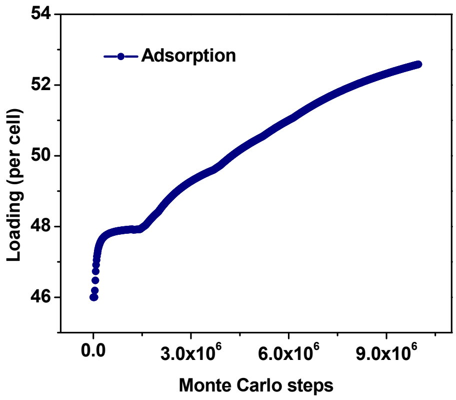


**Figure S29.** Simulated PFOA adsorption isotherm of PCN-999 in ambient condition.

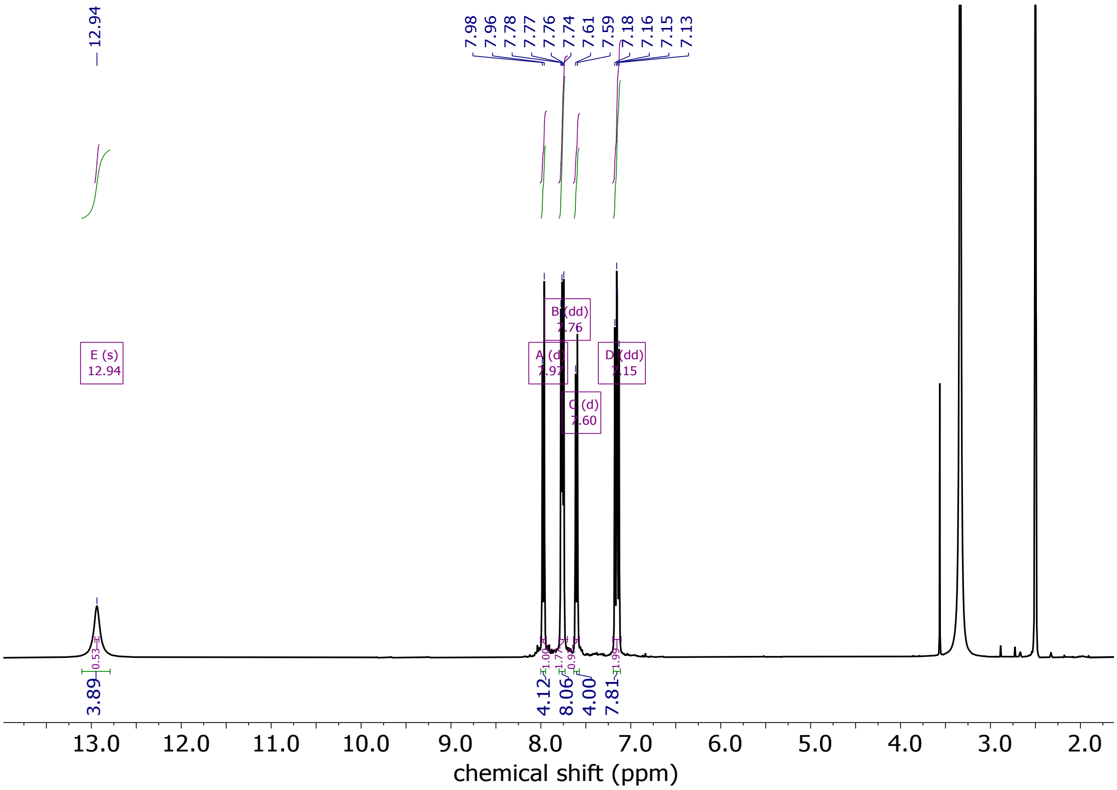


**Figure S30.** $^{1}$H NMR spectrum of L12.

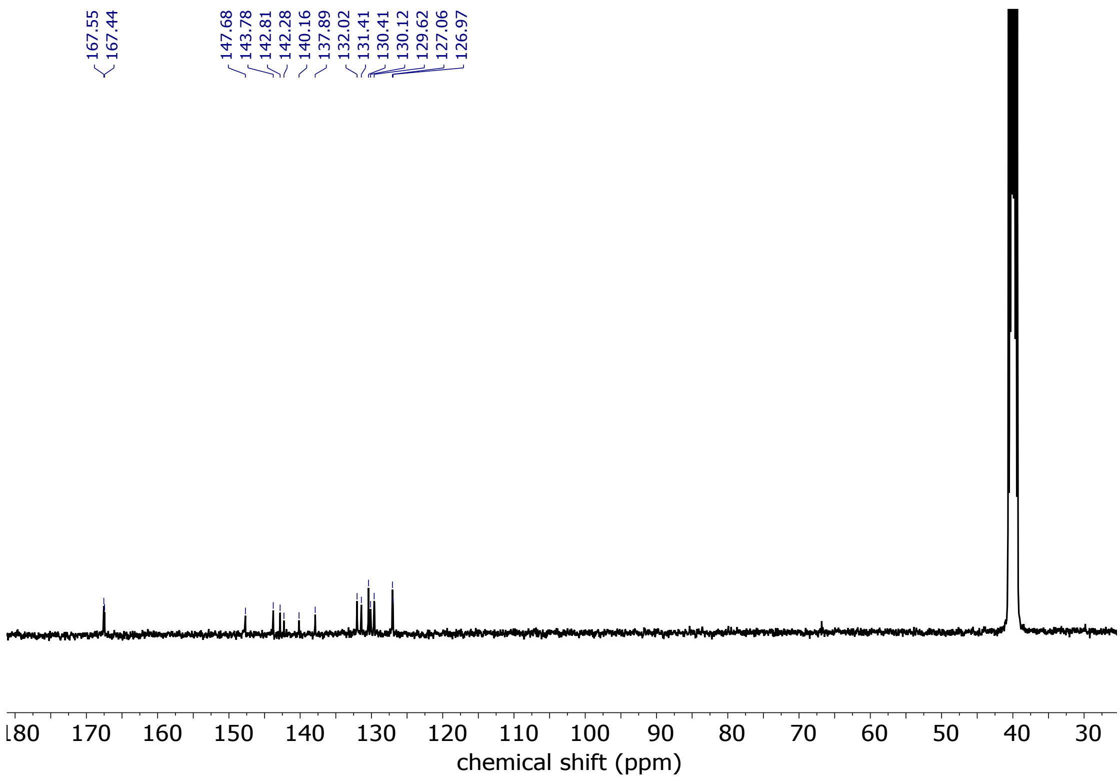


**Figure S31.** $^{13}C$ NMR spectrum of L12.

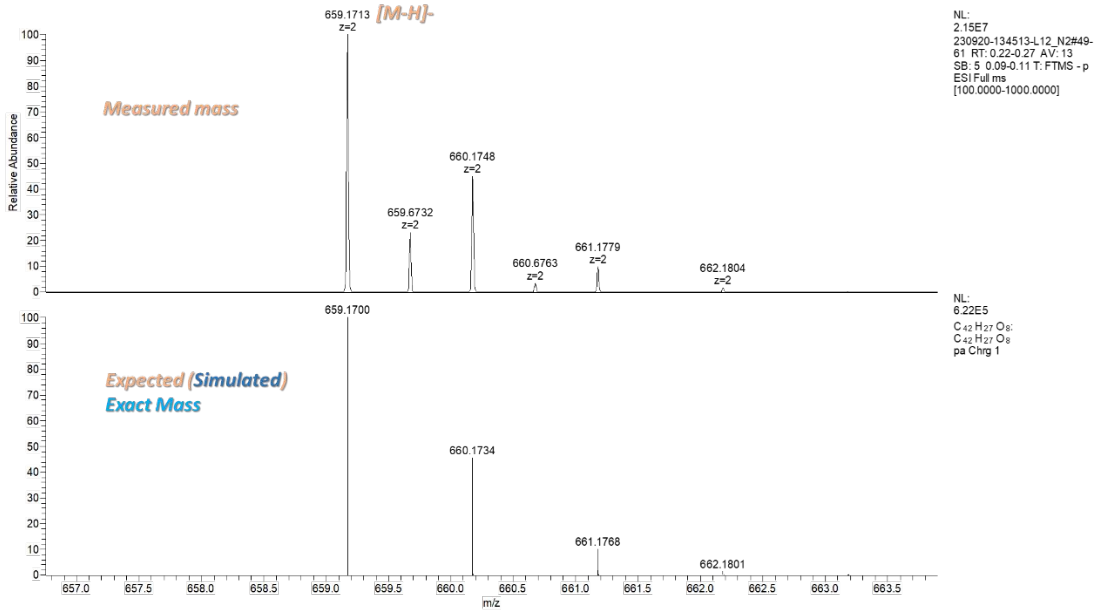


**Figure S32.** Mass spectrum of L12.

**Table S1.** Crystallographic data and structural refinement summary.

| Identification code | PCN-999 | PFOA@PCN-999 |
|---|---|---|
| CCDC | 2304331 | 2304332 |
| Empirical formula | $C_{169}H_{96}O_{80}Zr_{15}$ | $C_{1206}H_{606}F_{324}O_{480}Zr_{90}$ |
| Formula weight | 4774.75 | 37140.34 |

| | | |
|---|---|---|
| Temperature/K | 100.0 | 110.0 |
| Crystal system | orthorhombic | orthorhombic |
| Space group | *Cmmm* | *Cmmm* |
| $a$/Å | 27.991(2) | 28.546(2) |
| $b$/Å | 66.287(5) | 66.369(4) |
| $c$/Å | 29.179(2) | 29.682(3) |
| $\alpha$/° | 90 | 90 |
| $\beta$/° | 90 | 90 |
| $\gamma$/° | 90 | 90 |
| Volume/Å$^3$ | 54141(7) | 56235(8) |
| $Z$ | 4 | 0.666667 |
| $\rho_{calc}$ g/cm$^3$ | 0.586 | 0.731 |
| $\mu$/mm$^{-1}$ | 2.542 | 2.659 |
| $F$(000) | 9400.0 | 12132.0 |
| Crystal size/mm$^3$ | 0.2 × 0.2 × 0.001 | 0.1 × 0.1 × 0.1 |
| Radiation | Cu Kα (λ = 1.54184) | CuKα (λ = 1.54178) |
| 2$\theta$ range for data collection/° | 4.574 to 134.112 | 5.866 to 133.188 |
| Index ranges | $-25 \le h \le 33$, $-79 \le k \le 60$, $-34 \le l \le 27$ | $-32 \le h \le 33$, $-78 \le k \le 78$, $-35 \le l \le 35$ |
| Reflections collected | 176131 | 274896 |
| Independent reflections | 25058 [$R_{int}$ = 0.1997, $R_{sigma}$ = 0.0925] | 26008 [$R_{int}$ = 0.1421, $R_{sigma}$ = 0.0630] |
| Data/restraints/parameters | 25058/0/632 | 26008/643/858 |
| Goodness-of-fit on F$^2$ | 1.179 | 1.394 |
| Final R indexes [I>=2σ (I)] | $R_1$ = 0.1756, w$R_2$ = 0.3733 | $R_1$ = 0.1317, w$R_2$ = 0.3474 |
| Final R indexes [all data] | $R_1$ = 0.2358, w$R_2$ = 0.4211 | $R_1$ = 0.2004, w$R_2$ = 0.4279 |
| Largest diff. peak/hole / e Å$^{-3}$ | 5.12/-2.75 | 4.65/-1.30 |

**Table S2**. PFOA adsorption capacity of different reported adsorbents.

| Adsorbent | PFOA adsorption capacity (mg/g) | Reference |
|---|---|---|
| MIL-101-Cr | 460 | 3 |
| MIL-101-Cr-$NH_2$ | 290 | 3 |
| MIL-101-Cr-$NMe_2$ | 493 | 3 |
| MIL-101-Cr-DMEN | 534 | 3 |
| MIL-101-Cr-QDMEN | 754 | 3 |
| ZIF-7 | 22 | 4 |
| ZIF-8 | 177 | 4 |
| ZIF-L | 244 | 4 |
| UiO-66 | 388 | 5 |

| | | |
|---|---|---|
| UiO-66-F4 | 467 | 5 |
| UiO-67 | 743 | 6 |
| MIL-125-$NH_2$ | 42 | 7 |
| MIL-100-Fe | 427 | 8 |
| MIL-101-Fe | 490 | 8 |
| MIL-96-RHPAM2 | 340 | 9 |
| NU-1000 | 507 | 10 |
| F-MOF | 420 | 11 |
| DUT-5-2 | 92 | 12 |
| DFB-CDP | 33 | 13 |
| MWNTs | 140 | 14 |
| FCX4-P | 189 | 15 |
| AC | 53 | 16 |
| All-silica Beta (β) | 371 | 17 |
| PCN-999 | 1089 | This work |

**Reference**

[1] Dolomanov, O. V., Bourhis, L. J., Gildea, R. J., Howard, J. A. K., and Puschmann, H. *J. Appl. Cryst.* **42**, 339-341 (2009).

[2] Sheldrick, G. M. *Acta Crystallogr. A* **64**, 112-122 (2008).

[3] Liu, K., Zhang, S., Hu, X., Zhang, K., Roy, A., and Yu, G. *Environ. Sci. Technol*. **49**, 8657-8665 (2015).

[4] Chen, M. J., Yang, A. C., Wang, N. H., Chiu, H. C., Li, Y. L., Kang, D. Y., and Lo, S. L. *Microporous and Mesoporous Mater*. **236**, 202-210 (2016).

[5] Sini, K., Bourgeois, D., Idouhar, M., Carboni, M., and Meyer, D. *New J. Chem*. **42**, 17889-17894 (2018).

[6] Sini, K., Bourgeois, D., Idouhar, M., Carboni, M., and Meyer, D. *Mater. Lett*. **250**, 92-95 (2019).

[7] Ozdemir, O. K., and Ornstein, J. M. *TechConnect Briefs*, 213-216 (2019).

[8] Yang, Y., Zheng, Z., Ji, W., Xu, J., and Zhang, X. *J. Hazard. Mater*. **395**, 122686 (2020).

[9] Mohd Azmi, L. H., Williams, D. R., and Ladewig, B. P. *Chemosphere* **262**, 128072 (2021).

[10] Li, R., Alomari, S., Stanton, R., Wasson, M. C., Islamoglu, T., Farha, O. K., Holsen, T. M., Thagard, S. M., Trivedi, D. J., and Wriedt, M. *Chem. Mater*. **33**, 3276-3285 (2021).

[11] Ma, S.-Y., Wang, J., Fan, L., Duan, H.-L., and Zhang, Z.-Q. *J. Chromatogr. A* **1611**, 460616 (2020).

[12] Hu, Y., Guo, M., Zhang, S., Jiang, W., Xiu, T., Yang, S., Kang, M., Dongye, Z., Li, Z., and Wang, L. *Micropor. Mesopor. Mater*. **333**, 111740 (2022).

[13] Xiao, L., Ling, Y., Alsbaiee, A., Li, C., Helbling, D. E., and Dichtel, W. R. *J. Am. Chem. Soc*. **139**, 7689-7692 (2017).

[14] Li, X., Zhao, H., Quan, X., Chen, S., Zhang, Y., and Yu, H. *J. Hazard. Mater*. **186**, 407-415 (2011).

[15] Shetty, D., Jahovic, I., Skorjanc, T., Erkal, T. S., Ali, L., Raya, J., Asfari, Z., Olson, M. A., Kirmizialtin, S., Yazaydin, A. O., and Trabolsi, A. *ACS Appl. Mater. Interfaces* **12**, 43160-43166 (2020).

[16] Zhang, D., Luo, Q., Gao, B., Chiang, S.-Y. D., Woodward, D., and Huang, Q., *Chemosphere* **144**, 2336-2342 (2016).

[17] Bergh, M. V. den, Krajnc, A., Voorspoels, S., Tavares, S. R., Mullens, S., Beurroies, I., Maurin, G., Mali, G., and DeVos, D. E. *Angew. Chem. Int. Ed*. **59**, 14086-14090 (2020).
